# Recursive mentalizing and public salience in the perception of common knowledge

**DOI:** 10.1073/pnas.2536294123

**Published:** 2026-08-12

**Authors:** Julian De Freitas, Yuhui Huang, Steven Pinker

**Affiliations:** ^a^Marketing Unit, Harvard Business School, Cambridge, MA 02163; ^b^ Department of Psychology, Harvard University, Cambridge, MA 02138

**Keywords:** coordination, common knowledge, theory of mind, mentalizing, recursion

## Abstract

Common knowledge—what everyone knows that everyone knows, ad infinitum—is theoretically required for coordination. But a finite mind cannot represent infinitely embedded propositions, and even a few are cognitively taxing. We propose that people perceive common knowledge by combining a capacity-limited ability for recursive mentalizing with an intuitive sense of public salience: When an event is jointly and openly witnessed, they infer that recursively embedded beliefs are licensed. We probed participants’ attributions of embedded beliefs to characters in fictitious vignettes. Study 1 presented narratives in which the information available to characters was either asymmetrical, reciprocal, or public. Participants indicated their confidence in the characters’ beliefs with 0, 2, 4, 6, and 18 embeddings (the latter judged indirectly). Ratings showed i) a decline with number of embeddings, reflecting cognitive load, ii) modest overattribution of embedded beliefs in the nonpublic conditions, iii) far higher attribution of embedded beliefs in the public conditions, and iv) high indirect judgments that arbitrarily deep beliefs were justified. Study 2 replicated these findings with animated videos in which the characters’ information was private, reciprocal, doubly reciprocal, or public. Together, the studies indicate that people attribute common knowledge to observers of a publicly salient event, while being susceptible to capacity-driven recursion collapse and heuristic extrapolation (false embedded beliefs). This can explain how humans achieve coordination from sparse cues, yet sometimes miscoordinate when publicness is absent or embedding grows complex.

## Significance

Coordinating actions requires common knowledge: not just knowing something, but knowing that others know it, and so on. This seems impossible for finite minds, yet humans coordinate on scales from couples to countries. We propose people achieve coordination by tracking a few layers of who knows what who else knows and by treating publicly witnessed events as licensing implicit common knowledge. In two studies, participants inferred that public events justified arbitrarily deep mutual understanding, though they sometimes overextended or confused knowledge states. This mix of valid and faulty reasoning helps explain how humans achieve large-scale coordination while also being prone to missteps, from everyday faux pas to collective dilemmas such as bubbles, bank runs, panic buying, and strategic miscalculations.

Two or more people, say, Ed and Sally, are said to have common knowledge when Ed knows something, Sally knows it, Ed knows that Sally knows it, Sally knows that Ed knows it, and so on, ad infinitum (1–4). Common knowledge crucially differs from private knowledge (where Ed and Sally each know something but neither knows the other knows it) because it is necessary for coordination: two or more people making arbitrary but mutually beneficial choices. Human social life requires coordination on many scales, including everyday rendezvous and divisions of labor, social relationships such as friendship and authority, conventions such as language, currency, and driving side, and institutions like corporations and governments (3, 5–8). Each requires common knowledge, at least tacitly: A driver must know not just that driving on the right is the norm but that other drivers know it, too. Often this tacit knowledge takes the form of respecting a convention, where a coordination equilibrium has been stipulated or entrenched within a community, as in traffic laws or a national language. But spontaneous, flexible coordination is also a recurring feature of social life.

Despite the ubiquitous requirement for common knowledge in human affairs, how individual human minds perceive and mentally represent it remains obscure (4, 7, 9–12). A finite brain cannot represent an infinite list of recursive propositions, that is, those containing another proposition (a formulation of common knowledge sometimes called “iterate”). Conceivably people entertain a finite list together with the mental equivalent of “and so on,” “ad infinitum,” or “….” People are certainly capable of recursive mentalizing (thinking about what other people think that other people think, and so on), since it is essential to everyday social cognition, such as in persuading, detecting deception, correcting misimpressions, and following fictional plots. Yet recursive propositions with more than two or three embeddings are notoriously difficult for people to process, presumably because they overload or overwrite the mental “stack” which keeps track of the embedding (7, 13–16).

**Fig. 1.**
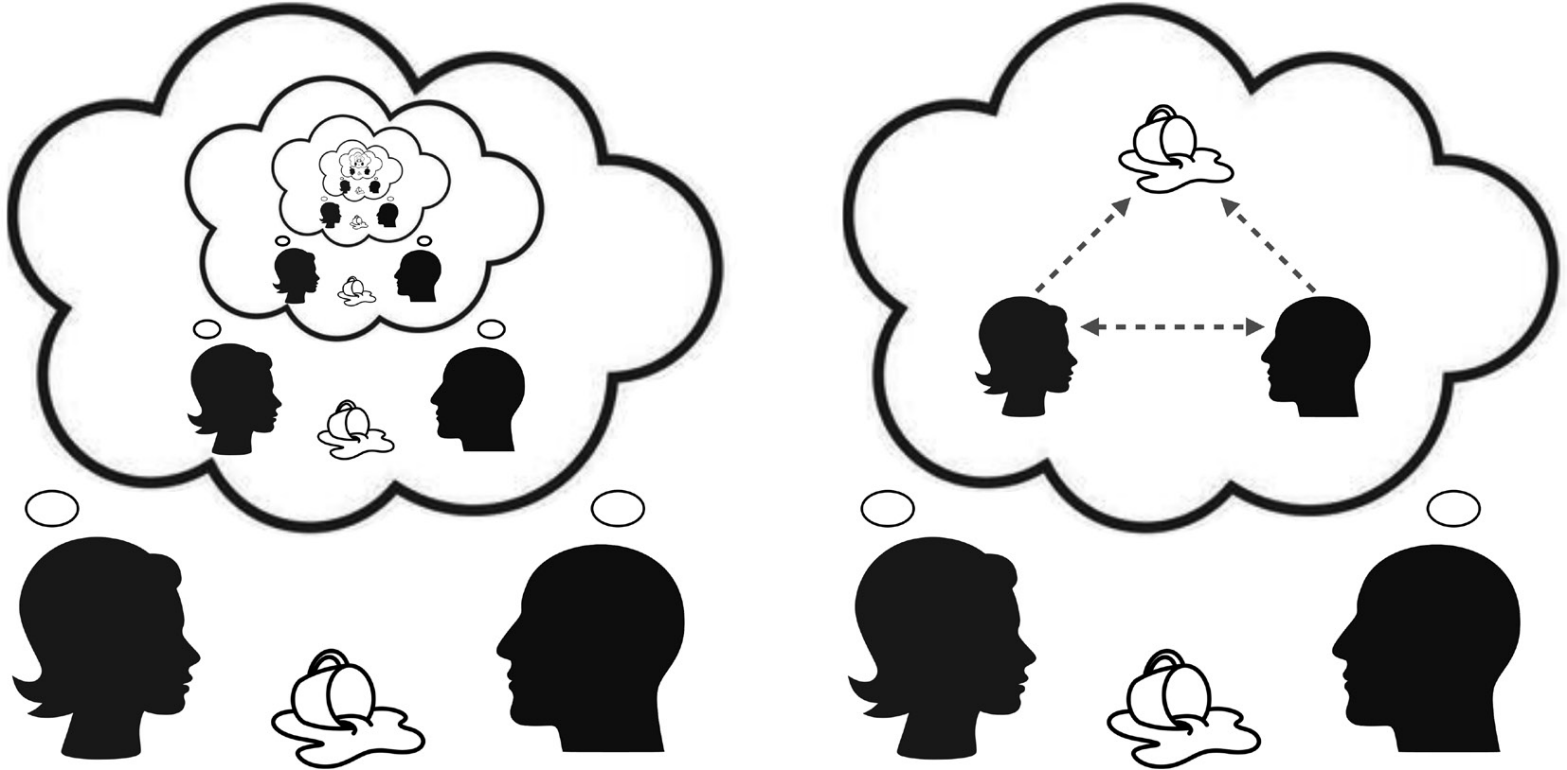
Two possible mental representations of common knowledge. *Left*: Recursive mentalizing. *Right*: The intuition that an event is publicly salient, self-evident, or “out there.”

These puzzles have led to the hypothesis that people represent common knowledge in some finite (or “fixed point”) representation. The simplest version would be the intuition that something is self-evident, publicly salient, or “out there” (8, 10, 12, 17). When an event is witnessed in circumstances in which another witness to it is witnessed [a situation of joint attention (18)], in principle common knowledge of the event may be inferred, and from that knowledge recursive beliefs of any finite depth may be deduced when needed. The two kinds of mental representation are caricatured in Fig. 1.

Note that for an event to be common knowledge, it cannot be merely salient, but must be publicly salient. That is, the public nature of the event—its visibility to others, and the visibility of the visibility—must itself be salient. The residents of an apartment building, for example, who look through cracks in their drapes and see an obvious act of vandalism in the courtyard have only private knowledge, not common knowledge, of the act. A commonly known event thus by definition includes awareness of that event, a kind of circularity or reflexiveness which may be captured in a finite self-referential formula like (*n*): Ed and Sally know *x,* and Ed and Sally know *n*,” or less formally, “Ed and Sally both know about the event, and all this is something they both know.”

Publicly salient events are sufficient to enable coordination even without recursive mentalizing, such as when simple organisms like coral evolve to use the full moon as a cue to synchronize release of gametes into the ocean. But humans figure out how to coordinate in countless evolutionarily novel activities, and presumably this depends on their inferring that publicly salient events entail recursive states of knowledge, including common knowledge. For example, a prominent advertisement for a new technology standard or internet marketplace reassures viewers that other viewers are aware of it, and so on, enabling widespread adoption (5).

Crucially, although a public event licenses an inference of recursive beliefs, including common knowledge, the converse is not true: No finite set of recursive belief states logically entails common knowledge (8, 12). In a classic example, two isolated army divisions would defeat an enemy if both attacked simultaneously but would be routed if one attacked alone. For a general to confidently send his division into battle, he must know not just that his counterpart is aware of his intention, but that the counterpart is aware of his knowledge of the counterpart’s awareness, and so on ad infinitum. In some circumstances, though, extrapolating from finite levels of awareness to common knowledge is a reasonable guess. For example, a child taught a novel word by an adult or peer may infer that it will be understood by any English speaker, even though in principle it could be proprietary to a family idiolect (19).

This paper proposes that humans perceive a state of common knowledge by an interplay between two mental processes: a capacity-limited ability for recursive mentalizing and an intuitive sense of public salience. The main prediction is that people justifiably infer that a public event leads to recursive knowledge of arbitrary depth. That is, they should infer not just that everyone in the audience is aware of the event, but aware that everyone is aware, and aware that everyone is recursively aware.

At the same time, people may be vulnerable to two kinds of error: recursion collapse, in which they fail to keep track of multiply embedded knowledge states, and recursion extrapolation, in which they mistakenly project private knowledge (or finitely embedded knowledge) into common knowledge. That would be a vicarious version of the “curse of knowledge,” the fallacy that what is known to oneself is known to everyone (20–22).

Past research has shown that people are indeed far likelier to take public announcements as opportunities to coordinate than messages which convey finite levels of embedded knowledge (6, 23–25). In one set of studies, people imagined themselves in the classic game-theoretic situation of a Stag Hunt, where two incommunicado hunters must choose whether to take a chance at meeting up to fell large quarry or to safely pursue smaller quarry individually; only common knowledge makes it rational to choose coordination (24, 26, 27). The participants were likelier to choose to coordinate when the presence of the large quarry was conveyed by a public smoke signal than by a sequence of one-way messages (and similarly in other scenarios exemplifying the game).

Though these studies show that publicness can lead to coordination, they do not prove that the connection is mediated by recursive mentalizing: As with the coral, the public event could directly trigger an urge to coordinate. Somewhat more relevant were studies of another public signal, blunt speech: People are likelier to attribute states of multiply embedded knowledge of a fraught proposition to conversational partners (“She knows that he knows that she knows … what he meant”) when one of them blurts it out than when he strongly implies it by innuendo (28).

The present two studies are more direct tests of whether people infer that public salience licenses an attribution of recursively embedded belief states, including common knowledge, and whether they navigate between these mental states in valid or invalid ways.^*^

Participants were shown scenarios in which an event is known by two people, but their knowledge about each other’s knowledge varies. Either they witnessed the event in each other’s presence (Public Information), one witnessed the event and the other came to know about it privately, unbeknownst to the first (Asymmetrical Information), or one witnessed it, the other also knew about it, and each privately knew the other knew about it (Reciprocal Information). We then probed their assessment of who knows what, with up to six levels of embedded knowledge about knowledge.

We could not, of course, literally test for common knowledge in the sense of an infinite series of ever-lengthening recursive propositions. But we probed it indirectly via a metacognitive approximation: whether people intuitively sensed that a recursive proposition of arbitrary depth, even one they could not possibly process, was licensed; in other words, whether they had “reason to believe” a multiply embedded proposition, even if cognitive limitations prevented them from explicitly believing it (29, 30). Participants chose among three commentators: one who believed the protagonists had beliefs embedded to an arbitrary depth (as common knowledge would imply), one who believed they did not, and a third who believed it was impossible to know.

In each case, we compared participants’ performance to an Ideal Observer, one whose judgments tracked the information about knowledge states presented in the vignette, and who makes only the logically licensed inference from publicness to recursion, without either collapsing embedded knowledge states downward or extrapolating them upward.

## Results

In Study 1, participants read scenarios in which a salient event was either jointly witnessed by two protagonists in each other’s presence (Public), one learned about it without the other’s knowledge (Asymmetrical), or each secretly knew of the other’s knowledge (Reciprocal). For example, in one of the Public Information scenarios, both Edward and his neighbor Sally witnessed a car crash directly in front of them, whereas in the corresponding Asymmetrical Information scenario, Sally learned of the crash from a roommate who also told her that Edward witnessed it. After each scenario, participants rated their confidence in attributing increasingly embedded beliefs to the characters (0, 2, 4, and 6 knowledge embedding levels).

**Fig. 2.**
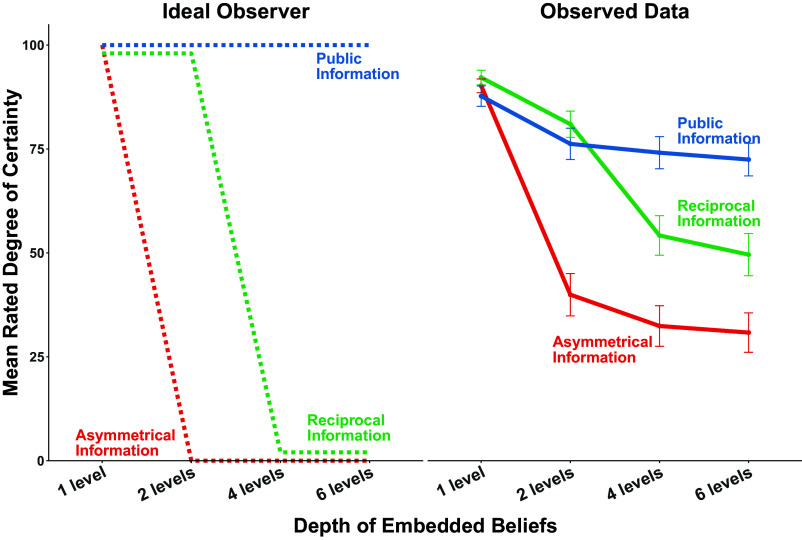
*Left*: Degree of certainty in an ideal observer (one who attributes recursive beliefs only to observers of public events) in beliefs with different depths of embedding. *Right*: Mean certainty ratings by the participants of Study 1.

The two panels in Fig. 2 plot degree of confidence in beliefs with varying levels of embedding for each of the three information conditions, averaged over scenarios. The left panel shows the judgments of a hypothetical ideal observer in this study: one who infers common knowledge (including finitely embedded beliefs) from a salient public event, but who neither spuriously collapses embedded beliefs into simpler ones, nor extrapolates them into more deeply embedded ones. The right panel shows the data from our participants.

A linear mixed-effects regression on the ratings, with the predictors Information Condition and Embedding Depth, and random intercepts for Participant, Vignette, and Presentation Order, confirms that mean ratings varied significantly with Embedding Depth (*P* < 0.001). There was no main effect of Information Condition (*P* = 0.396), but, as predicted, there was an interaction between Information and Embedding Depth (*P* < 0.001). Pairwise comparisons for each level of embedding within an Information condition confirm visual impressions: Pairs of points with nonoverlapping error bars were significantly different (all *P*s < 0.001).

The pattern of the interaction suggests that participants’ judgments reflect a composition of three effects. First, the participants attributed overly embedded beliefs to the protagonists in circumstances where only Asymmetrical or Reciprocal beliefs were justified. This is seen in the lowermost five points, which should be at the floor. Presumably this reflected a tendency to extrapolate simple states of belief to higher levels (a vicarious curse of knowledge), though it could partly reflect a compression of their use of the full rating scale.^†^

The second effect, seen in all three curves, is a decline in confidence with degrees of embedding, even across ranges where an ideal observer’s judgments would be pinned at the ceiling or floor. Presumably this occurred because more embedded propositions are harder to keep in mind, and participants were less confident in attributing or denying them to the characters.

Of greatest interest is the third effect: the pattern one would expect in an ideal observer, though attenuated. With a public event, where ideal judgments should be pinned at the ceiling, we see that participants were only a bit less confident in attributing two, four, and six levels of embedded knowledge to the characters (76.2%, 74.1%, and 72.5%, respectively) than knowledge about the event itself (87.7%). This was not a symptom of across-the-board overattribution of belief embedding, since they correctly judged that characters entitled only to asymmetrical knowledge were far less likely to have 2nd, 4th, and 6th-order beliefs and that characters entitled only to reciprocal knowledge were less likely to have 4th- and 6th-order beliefs.

The extent to which the data are well fitted by the latter two effects may be assessed by a regression with one predictor capturing a linear decline across increasing depth of embedding (reflecting cognitive load), another consisting of weights corresponding to the ideal observer pattern, and a third capturing differences in the slopes of the linear decline for the different knowledge conditions (if the effects of cognitive load are modulated by the different kinds of information). The ideal observer and linear decline contrasts were moderately correlated (r = 0.53), and the ideal observer and different slopes contrasts were modestly correlated (r = 0.28). Together the three variables accounted for 90.5% of the variance across the 12 condition means (ideal observer: 37.4%; linear decline: 4.9%; slope difference: 0.6%). Across participants, the variables account for 30.5% of the variance, and each of the first two predictors uniquely captured a significant portion (ideal observer: 12.6%, *P* < 0.001; monotonic decline: 1.6%, *P* < 0.001; slope difference: 0.2%, *P* = 0.166).

### Metacognitive Probe.

If people naturally infer common knowledge from public salience, and if this inference penetrates their conscious awareness, they should feel it is justifiable for the protagonists to have any number of nested beliefs, but only in the Public Information Condition.

**Fig. 3.**
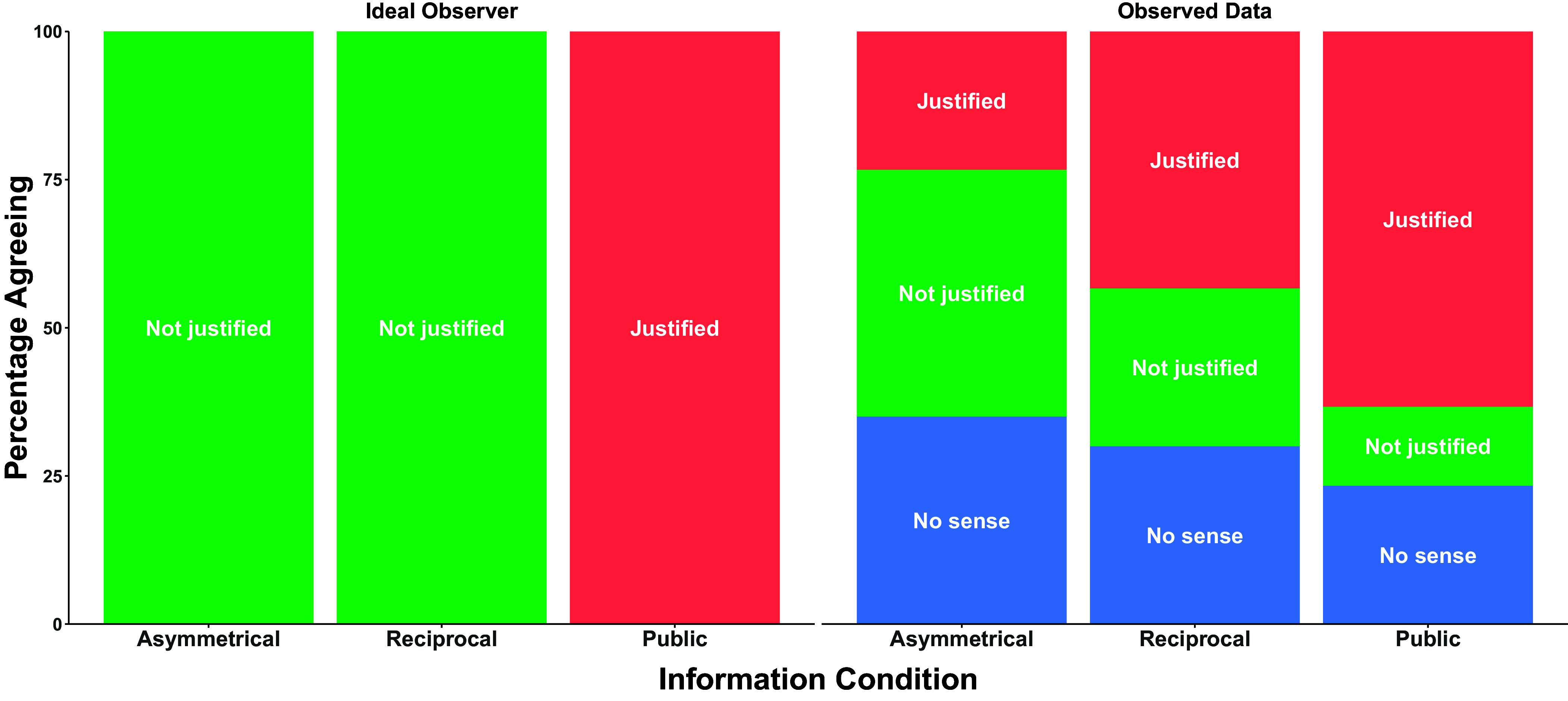
Agreement with attribution of arbitrarily embedded beliefs in different conditions of Information by an Ideal Observer who infers common knowledge only from Public Information (*Left*) and by the participants in Study 1.

Fig. 3 shows that participants were not exactly ideal observers. They illicitly felt it justifiable to attribute arbitrarily embedded beliefs to a character who had only Asymmetric Information 23.3% of the time, and in the other direction, they failed to conclude that it was justifiable to attribute such beliefs to the character who had public information 13.3% of the time. They did, however, show a strong tendency toward the ideal pattern, attributing the deeply embedded belief to the observer of a public event a majority of the time, 63.3%, far more than in the other information conditions (the distribution among the three judgments differed across the Information conditions ($\chi^{2}=21.33$, *P* < 0.001).

We conclude that through all the noise in people’s reflecting on construals of incomprehensibly embedded beliefs there is a signal of ideal inference from public salience to recursive knowledge.

Study 2 extends the inquiry from verbal descriptions to video depictions, to address the possibility that people have difficulty only in perceiving recursive *linguistic expressions*, not the mental states on which they are based. [This suspicion arises from the finding that children can recognize false beliefs depicted in actions years before they recognize them described in verbal narratives (31, 32).] The study also extends the range of information conditions to the simpler case of Private Information (in which two observers know of an event, but neither knows the other knows) and to the more complex case of Doubly Reciprocal Information (where each knows the other knows, and each knows that, while still falling short of common knowledge). For example, in one set of videos, two security guards saw a coffee spill within direct view of each other (Public Information), or they each saw the spill while hidden behind a bookshelf (Private Information), or they saw in surveillance footage that their witnessing of the incident had itself been espied by another guard (Doubly Reciprocal Information). As in Study 1, after seeing each vignette, participants rated their confidence that the characters held beliefs about each other’s beliefs about the event with multiple depths of embedding (0, 2, 4, and 6 levels). This design thus provides a further test of whether people correctly infer common knowledge from observation of public events, and also whether they sometimes incorrectly infer it by extrapolating from deeply embedded but finite knowledge states.

**Fig. 4.**
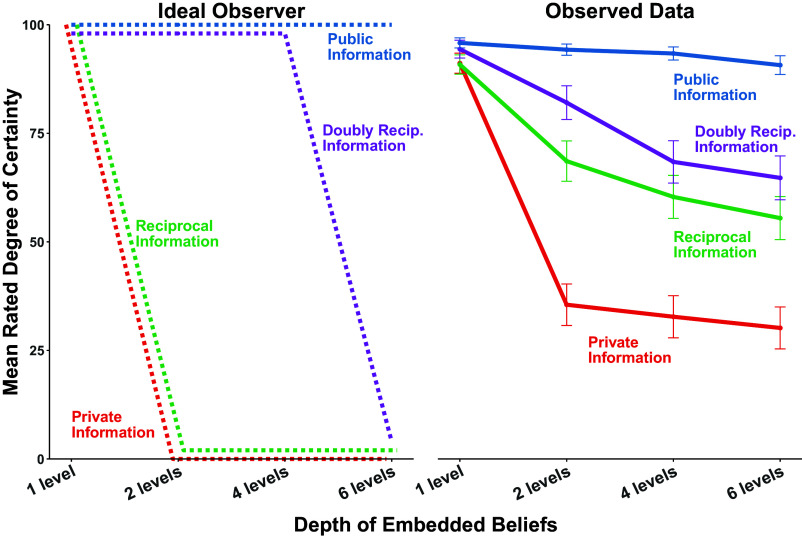
*Left*: Degree of certainty by an ideal observer in attribution of embedded beliefs in different conditions of information. *Right*: Mean degree of certainty ratings by the participants of Study 2.

Fig. 4 plots mean degree of confidence against number of embeddings for each information condition by a hypothetical ideal observer and as rated by our participants. Note that because there was no probe for 1 embedded belief, which would have distinguished Private from Reciprocal Information, the predictions for those two conditions are identical.^‡^

A linear mixed-effects regression on the ratings, with the predictors Information Condition and Embedding Depth, and random intercepts for Participant and Presentation Order, confirms that mean ratings varied significantly with Embedding Depth (*P* < 0.001). There was no main effect of Information Condition (*P* = 0.391), but, as predicted, there was an interaction between Information and Embedding Depth (*P* < 0.001). Pairwise comparisons for each level of embedding within an Information condition confirm visual impressions: Pairs of points with nonoverlapping error bars were significantly different (all *P*s < 0.001).

As in Study 1, participants showed a tendency to overattribute embedded beliefs with nonpublic information, in this case even when the information was Private (30.2 to 35.5%). And as before, we see declines in confidence with increasing depth of embedding, even in ranges where an ideal observer would show no difference, with the slope of the decline depending on the information condition (presumably due to cognitive load). We also see, importantly, a strong signal of Ideal recursive belief attribution: With Public Information, participants attributed beliefs with 2, 4, and 6 embeddings almost as confidently as knowledge of the event itself, whereas with Private Information, attribution of embedded belief was far lower (though as noted, not zero).

As before, we confirmed the hypothesized combination of effects of degree of embedding (reflecting cognitive load) with an ideal observer pattern (reflecting inference from publicness to common knowledge) in a regression with three predictors. Across the 16 means, the three predictors together accounted for 74.4% of the variance (ideal observer = 39.3%; linear decline, 0.18%; slope difference, 0.88%). Across participants, the three predictors collectively accounted for 29.2% of the variance (ideal observer: 15.4%, *P* < 0.001; linear decline: 0.07%, *P* = .346; slope difference: 0.34%, *P* = 0.036).

### Metacognitive Probe.

**Fig. 5.**
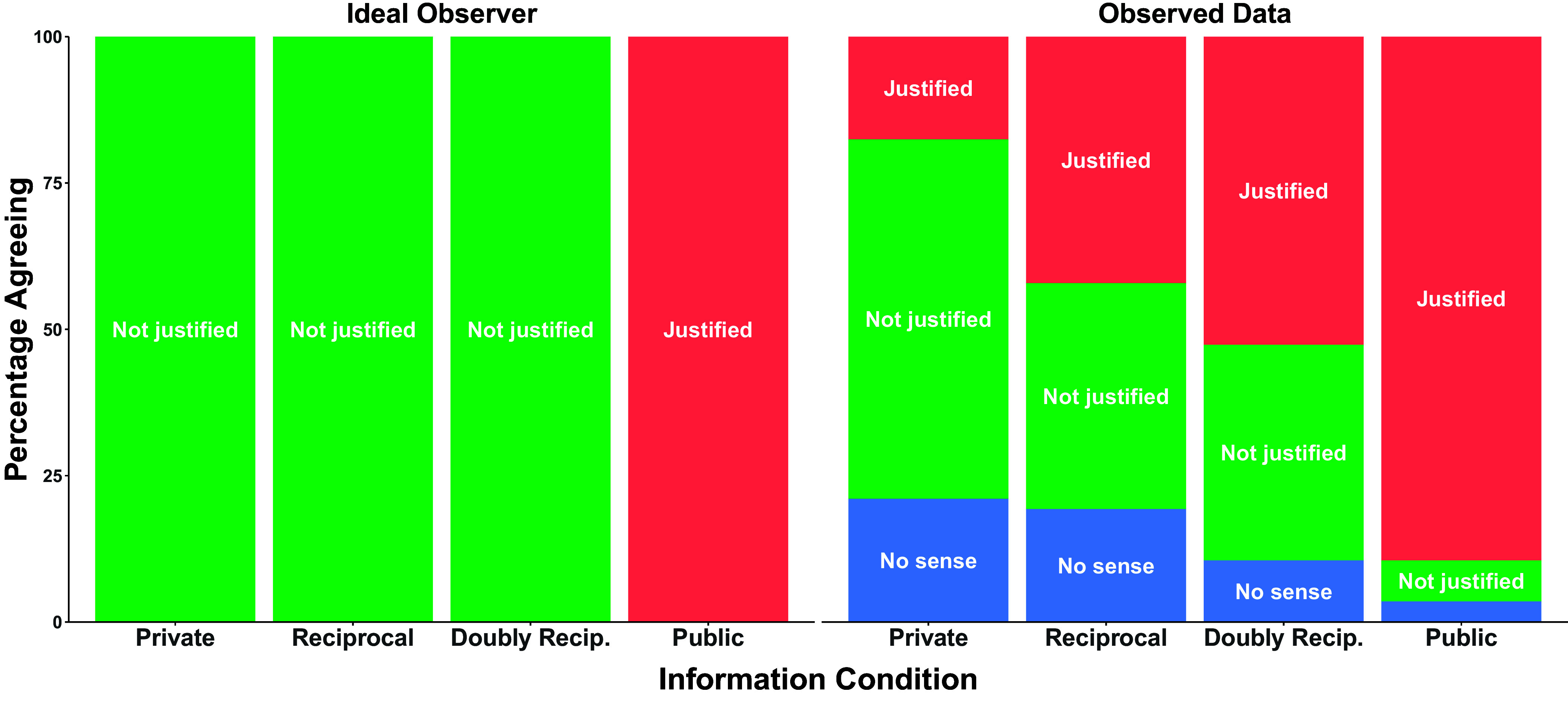
Agreement with attribution of arbitrarily embedded beliefs by an Ideal Observer (*Left*) and by the participants in Study 2 (*Right*).

The ideal and observed degree of agreement with attributions of a belief with 18 embeddings is shown in Fig. 5. Contingency tests confirm that the proportions differ by Information condition ($\chi^{2}=62.30$, *P* < 0.001). Even more than in Study 1, participants attributed arbitrarily embedded beliefs to an observer of a public event: 89.5% agreed with a commentator who endorsed this attribution, compared to only 17.5% who agreed in the case of a private observer. And as in Study 1, this signal of normative inference to common knowledge was mingled with a graded overattribution of highly embedded beliefs when there was only private and reciprocal information and they should have been zero. Again, this suggests that people have some tendency to extrapolate finite embedded beliefs to common knowledge, a vicarious curse of knowledge.

## General Discussion

We asked how people perceive a state of common knowledge, necessary for coordination on scales large and small, when it would seem to require, impossibly, mentally representing an infinite list of ever-more-complex recursive beliefs. We tested the possibility that people represent it as an intuition that the knowledge is self-evident or “out there,” inferred from a salient public event. We also tested whether people, exercising a distinct capacity-limited talent for recursive mentalizing, extrapolate from shallowly embedded beliefs to a tacit acceptance of more deeply embedded ones, all the way up to common knowledge—an inference that is not logically justified but sometimes a reasonable heuristic. By showing participants scenarios with events that are either publicly salient or observed under different conditions of the observer being observed, we concluded that people have a strong (though not automatic) tendency toward the first kind of inference (a public event implies common knowledge), and slight (though persistent) tendency toward the second (finitely embedded knowledge implies common knowledge).

This complex mix of frequent valid and occasional invalid inferences to common knowledge, together with confusions among levels of embedded knowledge, may help to resolve the paradox that finite human minds can enjoy the benefits of coordination while also being vulnerable to miscoordinations and the resulting social problems (5, 7). At smaller, personal scales, these may consist of faux pas and snafus such as inadvertently blurting out a secret, having a seemingly obvious implication go over one’s head, playing Alphonse-and-Gaston at a four-way stop sign, writing in opaque academic jargon, and being the sole applauder between movements of a sonata. At larger, collective scales, it may consist of pluralistic ignorance (when everyone assumes that everyone believes something, such as support for a regime, but no one actually believes it), commitment to an inferior technological standard (as in VHS vs. Betamax), misjudging an enemy’s resolve in an international game of chicken, or acting on rumors that reverberate into self-fulfilling bubbles, hoarding, panic buying, and bank runs.

## Materials and Methods

All studies were approved by Harvard’s Institutional Review Board (protocol IRB16-0158), and all participants provided informed consent. Data and code are available at Github (33).

In Study 1, we recruited 69 participants (mean age 36, 33% female) from Amazon Mechanical Turk who passed a simple attention screen. Each participant completed the survey once; no weighting or poststratification procedures were applied. We excluded 9 who failed basic comprehension checks about the topic of the story.

The study employed a mixed design: three kinds of Information (Asymmetrical, Reciprocal, Public) × 3 Scenarios (Visual Salience, Eye Contact, Auditory Salience) × 6 orders. The three scenarios were intended to capture distinct ways in which an event can be public: visible in each other’s presence, mutually acknowledged by eye contact, audible in each other’s presence.

Each participant read three scenarios, each with a different kind of information. The order of the scenarios was constant (Visual, Eye Contact, Auditory) while the order of information conditions varied; pairings and orders were counterbalanced across participants.

### Visual Salience.

Participants read:

Edward and his neighbor, Sally, share a garden in a medium-sized apartment complex. One day Edward is watering their garden, when a car crash occurs on the street right in front of the garden in which he is working.

The Asymmetrical Information condition continued:

Sally’s roommate notices the event from her window, and calls Sally to let her know about the crash and that Edward witnessed it.

If participants are ideal observers, inferring no more than what was stated and logically justified, they should conclude only that Edward and Sally each know about the crash and that Sally knows that Edward knows about it.

The Reciprocal Information condition continued with:

Sally’s roommate notices the event from her window, and calls Sally to let her know about the crash and that Edward witnessed it. She then goes outside to talk to Edward about the crash, and tells him all about her phone call with Sally.

An ideal observer would infer only that Edward and Sally each know and that each knows that the other knows.

In the Public Information condition, the second sentence in the story had both neighbors witnessing the event:

One day they are talking and watering their garden, when a car crash occurs on the street right in front of the garden in which they are working.

An ideal observer who inferred common knowledge from a publicly and mutually observed event could infer any number of arbitrarily embedded knowledge states.

### Eye Contact.

Participants read:

Edward and Sally are sitting across from each other on a train, when Edward notices a man on the other side of the train silently walk up to another passenger who is wearing a provocative t-shirt, say something to him, then give him the finger. While this is happening, Sally glances toward Edward and sees that he is watching the event.

In the Asymmetrical Information condition, the story continued:

However, Edward does not notice Sally do this.

An ideal observer would conclude that Edward and Sally each know about the confrontation and that Sally knows that Edward knows about it.

In the Reciprocal Information condition, it continued:

Edward immediately spots this out of the corner of his eye, but pretends not to notice her.

The ideal observer’s takeaway is that Edward and Sally each know about the confrontation, and each knows the other knows.

In the Public Information condition, the confrontation was described as occurring between the two friends, visible to both. An ideal observer can infer common knowledge.

### Auditory Salience.

The scenario began:

Sally barges into her bosses’ office and shouts in frustration, “Please tell Edward to get rid of his childish Looney Tunes cellphone ringtone. Yesterday his phone rang in front of an important client.”

In the Asymmetrical Information condition, it continued:

Just as Sally is uttering this sentence, Edward walks past the room and overhears her say this. However, Sally does not see Edward pass by.

Edward and Sally each know about Sally’s complaint (in the case of Sally, because she herself made it), and Edward knows Sally knows.

In the Reciprocal Information condition, it continued:

Just as Sally is uttering this sentence, Edward walks past the room and overhears her say this. Instead of continuing down the hallway, Edward hides outside the door and hears Sally say, “Oops, I just saw Edward and I think he heard everything.”

Edward and Sally each know about Sally’s complaint, and each knows the other knows.

In the Public Information condition, it continued,

Right at this point, Edward gets a call and his phone rings loudly, playing the Looney Tunes song.

Common knowledge may be inferred.

After reading each story, and with it still visible, participants rated four possible beliefs of Edward, with increasing depths of embedding, by adjusting a 100-point slider (1 = “Not certain at all,” 100 = “Completely certain”). To ease the cognitive demand, we varied the verbs of knowing (which reduced self-similarity), and broke the beliefs into separate sentences (which reduced the working memory load). In the visual salience scenario, for example, they were asked “Is Edward thinking….”

[0 embeddings (event itself):] “A car crash just happened.”

[2 embeddings:] “Sally knows I’m aware of the car crash.”

[4 embeddings:] “Sally knows I’m aware of the car crash. And she understands that I realize that she knows it.”

[6 embeddings:] “Sally knows I’m aware of the car crash. And she understands that I realize that she knows it, and also that I know that she knows this.”

Then, to estimate participants’ tacit attribution of common knowledge in the sense of a potentially infinite number of nested knowledge states, we gave them a metacognitive task that asked them to consider whether Edward believed a thought with 18 levels of embedding. In the visual salience scenario, it was:

Sally knows that I’m aware that she realizes that I understand that she recognizes that I know that she’s aware that I realize that she understands that I recognize that she knows that I’m aware that she realizes that I understand that she recognizes that I know that she understands that I’m aware of the car crash.

Since a direct rating of confidence in Edward’s belief in this patently incomprehensible sentence was inappropriate, we assessed their metacognitive judgment of whether the belief was justified (whether Ed had reason to believe it) by asking which of three commentators they agreed with. They were told that Art, Bart, and Hart all find the sentence “difficult to think about,” but “Art and Bart think this is irrelevant, since they still know the answer.”

Art’s sense is that no matter how many more “Edward knows that Sally knows…” clauses there are, Edward and Sally both know that they both know (and so on) that Edward is aware of the car crash.
  Bart’s sense is that no matter how many more “Edward knows that Sally knows…” clauses there are, Edward and Sally both do not know that they both know (and so on) that Edward is aware of the car crash.
  Hart, however, thinks there is no way to know the answer, since he has no sense at all of whether the sentence could be correct.

In Study 2, we recruited 59 participants (28 female) from Prolific and excluded two who failed attention checks. Each participant completed the survey once; no survey weighting or poststratification procedures were applied. The study employed a mixed design: four kinds of Information (Private, Reciprocal, Doubly Reciprocal, Public) × 24 orders. The four Information conditions were presented within subjects, order counterbalanced.

Participants watched a cartoon animation of an event in a library with a “No food or drink” sign posted. Sally and Edward are security guards.

In the Private Information condition, Sally and Edward are stationed in different aisles between book stacks with no line of sight between them. Sally and Edward both witness a student accidentally spilling coffee on a table, depicted by animated arrows extending from each one’s eyes to the spill, but neither can see the other. (Video: https://youtu.be/v3c9_kQz6SY.)

The Reciprocal Information condition was the same, except that Sally peers through a gap between the bookshelves and sees Edward staring at the coffee spill (shown by an animated arrow extending from her head to his). Unbeknownst to her, Edward then peers through a different gap and sees her looking at the spill, also depicted by an animated arrow. (Video: https://youtu.be/MCFgM_v2RJg.) Thus, each knows about the spill, and each knows the other knows.

In the Doubly Reciprocal Information Condition, this sequence was followed by the characters returning to separate offices and reviewing the security camera footage. Edward has a thought bubble, “Sally will report the coffee incident,” but then sees footage of Sally seeing him seeing the spill, and the thought bubble changes to an exclamation point. Sally undergoes the same sequence. (Video: https://youtu.be/XVjK8AmZk1E.) Now, Edward knows that Sally knows that Edward knows about the event, and vice versa.

In the Public Information Condition. Sally and Edward are in the open, each seeing the spill and seeing each other see it (depicted by four animated arrows). (Video: https://youtu.be/VXq-bAD6CiA).

After each condition, participants were told that the protagonists do not communicate with each other. As in Study 1, after watching each video, participants rated four first-person statements about Edward’s knowledge, with increasing numbers of embedded beliefs. This time we further disambiguated the question by replacing “it” in the Study 1 questions with the exact proposition. Thus, participants were asked “Is Edward thinking….”

[0 embeddings (event itself):] “Someone spilled a cup of coffee in an area where food and drink are prohibited.”
  [2 embeddings:] “Sally knows I’m aware of the coffee incident.”
  [4 embeddings:] “Sally knows I’m aware of the coffee incident. And she understands that I realize that she knows that I’m aware of the coffee incident.”
  [6 embeddings:] “Sally knows I’m aware of the incident. And she understands that I realize that she knows that I’m aware of the coffee incident, and also that I know that she knows this.”

They then answered the metacognitive probe with the 18-deep test sentence and three commentators.

## Supplementary Material

Appendix 01 (PDF)

## Data Availability

Survey response data are available in Github (33).

## Footnotes

^*^In this paper, we use “knowledge,” which technically refers only to justifiable true beliefs, to embrace credible beliefs, and “common knowledge” to embrace credible common belief [“common *p*-belief” (12)].

^†^It’s unlikely to consist entirely of rating-scale compression because the pattern of interaction differs qualitatively from that of an ideal observer: Reciprocal information (the more cognitively demanding condition) elicited greater embedded belief attribution than did asymmetric information.

^‡^Consistent with the findings in these studies, this very design flaw arose from of an instance of recursion extrapolation by the first and third authors, who mistakenly assumed that asymmetric information licensed two levels of embedded belief, until the error was spotted by the second author.

## References

[1] T. C. Schelling, The Strategy of Conflict (Harvard University Press, Cambridge, MA, 1960).

[2] R. Aumann, Agreeing to disagree. Ann. Stat. **4**, 1236–1239 (1976).

[3] D. K. Lewis, Convention: A Philosophical Study (Harvard University Press, Cambridge, MA, 1969).

[4] M. F. Friedell, On the structure of shared awareness. Behav. Sci. **14**, 28–39 (1969).

[5] M. S.-Y. Chwe, Rational Ritual: Culture, Coordination, and Common Knowledge (Princeton University Press, Princeton, NJ, 2001).

[6] J. De Freitas, K. Thomas, P. DeScioli, S. Pinker, Common knowledge, coordination, and strategic mentalizing in human social life. Proc. Natl. Acad. Sci. U.S.A. **116**, 13751–13758 (2019). 31253709 10.1073/pnas.1905518116 PMC6628641

[7] S. Pinker, When Everyone Knows that Everyone Knows...: Common Knowlege and the Mysteries of Money, Power, and Everyday Life (Scribner, New York, 2025).

[8] J. Geanakoplos, Common knowledge. J. Econ. Perspect. **6**, 53–82 (1992).

[9] H. Lederman, Uncommon knowledge. Mind **127**, 1069–1105 (2018).

[10] H. H. Clark, Using Language (Cambridge University Press, New York, 1996), p. xi, 432.

[11] J. Barwise, “Three views of common knowledge” in Proceedings of the Second Conference on Theoretical Aspects of Reasoning About Knowledge, M. Y. Vardi, Ed. (Morgan Kaufman, 1988), pp. 365–379.

[12] D. Monderer, D. Samet, Approximating common knowledge with common belief. Games Econom. Behav. **1**, 170–190 (1989).

[13] J. Perner, H. Wimmer, “John thinks that Mary thinks that...”: Attribution of second-order beliefs by five- to 10-year-old children. J. Exp. Child Psychol. **39**, 437–471 (1985).

[14] P. Kinderman, R. Dunbar, R. P. Bentall, Theory-of-mind deficits and causal attributions. Br. J. Psychol. **89**, 191–204 (1998).

[15] R. Nagel, Unraveling in guessing games: An experimental study. Am. Econom. Rev. **85**, 1313–1326 (1995).

[16] J. Mehta, C. Starmer, R. Sugden, The nature of salience: An experimental investigation of pure coordination games. Am. Econom. Rev. **84**, 658–673 (1994).

[17] R. J. Aumann, Interactive epistemology I: Knowledge. Int. J. Game Theory **28**, 263–300 (1999).

[18] M. Tomasello, M. Carpenter, J. Call, T. Behne, H. Moll, Understanding and sharing intentions: The origins of cultural cognition. Behav. Brain Sci. **28**, 675–691 (2005). 16262930 10.1017/S0140525X05000129

[19] G. Diesendruck, L. Markson, Children’s avoidance of lexical overlap: A pragmatic account. Dev. Psychol. **37**, 630–644 (2001). 11552759

[20] S. A. J. Birch, P. Bloom, The curse of knowledge in reasoning about false beliefs. Psychol. Sci. **18**, 382–386 (2007). 17576275 10.1111/j.1467-9280.2007.01909.x

[21] C. Camerer, G. Lowenstein, M. Weber, The curse of knowledge in economic settings: An experimental analysis. J. Polit. Econ. **97**, 1232–1254 (1989).

[22] S. Pinker, The Sense of Style: The Thinking Person’s Guide to Writing in the 21st Century (Penguin, New York, 2014).

[23] K. A. Thomas, J. De Freitas, P. DeScioli, S. Pinker, Recursive mentalizing and common knowledge in the bystander effect. J. Exp. Psychol. Gen. **145**, 621–629 (2016). 26913616 10.1037/xge0000153

[24] K. A. Thomas, P. DeScioli, O. S. Haque, S. Pinker, The psychology of coordination and common knowledge. J. Pers. Soc. Psychol. **107**, 657–676 (2014). 25111301 10.1037/a0037037

[25] K. A. Thomas, P. DeScioli, S. Pinker, Common knowledge, coordination, and the logic of self-conscious emotions. Evol. Hum. Behav. **39**, 179–190 (2018).

[26] J.-J. Rousseau, Discourse upon the Origin and Foundation of Inequality among Mankind (Oxford University Press, New York, 1994).

[27] B. Skyrms, The Stag Hunt and the Evolution of Social Structure (Cambridge University Press, New York, 2004).

[28] J. J. Lee, S. Pinker, Rationales for indirect speech: The theory of the strategic speaker. Psychol. Rev. **117**, 785–807 (2010). 20658853 10.1037/a0019688

[29] H. H. Clark, C. R. Marshall, “Definite reference and mutual knowledge” in Elements of Discourse Understanding, A. K. Joshi, B. L. Webber, I. A. Sag, Eds. (Cambridge University Press, New York, 1981), pp. 10–63.

[30] R. P. Cubitt, R. Sugden, Common knowledge, salience, and convention: A reconstruction of David Lewis’s game theory. Econ. Philos. **19**, 175–210 (2003).

[31] D. Butttelmann, M. Carpenter, M. Tomasello, Eighteen-month-old infants show false belief understanding in an active helping paradigm. Cognition **112**, 337–342 (2009). 19524885 10.1016/j.cognition.2009.05.006

[32] B. Knudsen, U. Liszkowski, 8-month-olds predict specific action mistakes through attribution of false belief, not ignorance, and intervene accordingly. Infancy **17**, 672–691 (2012). 32693489 10.1111/j.1532-7078.2011.00105.x

[33] J. De Freitas, Y. Huang, S. Pinker, Data and code for “Recursive mentalizing and public salience in the perception of common knowledge.” GitHub. https://github.com/Ethical-Intelligence-Lab/infinite_mind. Deposited 28 July 2026. 10.1073/pnas.2536294123 PMC13486529 42585001

[34] C. J. Clark , Prosocial motives underlie scientific censorship by scientists: A perspective and research agenda. Proc. Natl. Acad. Sci. U.S.A. **120**, e2301642120 (2023). 37983511 10.1073/pnas.2301642120 PMC10691350

