# Supplementary material for: Recursive mentalizing and public salience in the perception of common knowledge
Source: Proc Natl Acad Sci U S A. 2026 Aug 12;123(33):e2536294123. doi: 10.1073/pnas.2536294123 (PMC13486529; doi:10.1073/pnas.2536294123)
Supplement: Supplementary file 1 [file pnas.2536294123.sapp.pdf]

Study 1 Survey

Questions

CONSENT

Participation is voluntary
It is your choice whether or not to participate in this research. If you choose to participate, you may change your mind and leave the study at any time. Refusal to participate or stopping your participation will involve no penalty or loss of benefits to which you are otherwise entitled.

What is the purpose of this research?
The purpose of this research is to examine human visual performance and judgments. All data from this experiment are gathered for scientific purposes and will contribute to our eventual understanding of brain and visual function. These data may be published in scientific journals so that other researchers may have access to these data.

How long will I take part in this research?
Your participation will take approximately 10 minutes to complete.

What can I expect if I take part in this research?
As a participant, you will be asked to look at images presented on a video display and give responses with key presses or movements of a mouse pointing device. Your response may involve responding as quickly as you can, memorizing what you saw, making a judgment, or completing a questionnaire. You will also be asked to complete a demographics form.

What are the risks and possible discomforts?
If you choose to participate, the effects should be comparable to those you

would experience from viewing a computer monitor for 10 minutes and using a mouse or keyboard, e.g., eye fatigue. You are free to take breaks throughout the session. Some of the images and stories are mildly emotional, and some of the written stories are disgust-inducing.

Will I be compensated for participating in this research?
You will be compensated $0.25 for this study. You will still receive payment if you withdraw early.

If I take part in this research, how will my privacy be protected? What happens to the information you collect?
Your participation in this experiment will remain confidential, and your identity will not be stored with your data.

If I have any questions, concerns or complaints about this research study, who can I talk to?
The researcher for this study is Julian De Freitas who can be reached at 33 Kirkland St, Cambridge MA, 02138; defreitas@g.harvard.edu. The faculty sponsor is George A. Alvarez who can be reached at alvarez@wjh.harvard.edu.

- If you have questions, concerns, or complaints,
- If you would like to talk to the research team,
- If you think the research has harmed you, or
- If you wish to withdraw from the study.

This research has been reviewed by the Committee on the Use of Human Subjects in Research at Harvard University. They can be reached at 617-496-2847, 44-R Brattle Street, Suite 200, Cambridge, MA 02138, or cuhs@harvard.edu for any of the following:

- If your questions, concerns, or complaints are not being answered by the research team,

- If you cannot reach the research team,
- If you want to talk to someone besides the research team, or
- If you have questions about your rights as a research participant.

I consent

ATTENTION CHECKS

John is taller than Paul. Who is shorter?

- **John**
- **Paul**
- **Neither John nor Paul**
- **Both John an Paul**

What color is grass?

The fresh, uncut grass, not leaves or hay. Please make sure to select purple, so that we know you're paying attention.

- **Green**
- **Purple**

ATTENTION CHECKS 2

You failed one of the previous attention checks. Please pay closer attention!

Here are some more attention checks:

This is an attention check. Please set X at 15, and make it so Y is larger than X, and Z is larger than Y. Please also make sure that Y is evenly divisible by 10.

	0	10	20	30	40	50	60	70	80	90	100	
X												
Y												
Z												

How many fatal heart attacks have you had?

- 0
- 1
- 10
- 100
- 1000

INTRO

Welcome! You will read three different stories. Please read them **very carefully**, then answer 3 pages of short questions about each one.

1_1 - PRIVATE CRASH

Edward and his neighbor, Sally, share a garden in a medium-sized apartment complex. One day Edward is watering their garden, when a car crash occurs on the street right in front of the garden in which he is working.

Sally’s roommate notices the event from her window, and calls Sally to let her know about the crash and that Edward witnessed it.

Put yourself in Edward's shoes. Please rate how certain you are about each of the things that Edward might be aware of. Think carefully about each statement-- it may seem complicated at first, but most people find that when they take their time and think about each sentence in turn, the situation becomes clear. Please consider each one independently of the others.

What is Edward aware of?

1. A car crash just happened.

No, he definitely isn't aware of that — Yes, he definitely is aware of that

0 10 20 30 40 50 60 70 80 90 100

2. "Sally knows I'm aware of the car crash."

No, he definitely isn't aware of that | Yes, he definitely is aware of that

0 10 20 30 40 50 60 70 80 90 100

3. "Sally knows I'm aware of the car crash. And she understands that I realize that she knows it."

No, he definitely isn't aware of that | Yes, he definitely is aware of that

0 10 20 30 40 50 60 70 80 90 100

4. "Sally knows I'm aware of the car crash. And she understands that I realize that she knows it, and also that I know that she knows this."

No, he definitely isn't aware of that | Yes, he definitely is aware of that

0 10 20 30 40 50 60 70 80 90 100

Here's the story again:

Edward and his neighbor, Sally, share a garden in a medium-sized apartment complex. One day Edward is alone in the garden watering the

plants, when a car crash occurs on the street right in front of the garden in which he is working.

Sally's roommate notices the event from her window, and calls Sally right then to let her know about the crash and that Edward witnessed it.

Now, let's imagine whether Edward is thinking something really complicated:

"Sally knows that I'm aware that she realizes that I understand that she recognizes that I know that she's aware that I realize that she understands that I recognize that she knows that I'm aware that she realizes that I understand that she recognizes that I know that she understands that I'm aware of the car crash."

Imagine three people named Art, Bart and Hart. Art, Bart and Hart disagree about their understanding of this sentence. Although they all find it very difficult to think about, Art and Bart think this is irrelevant, since they still know the answer.

Art's sense is that, no matter how many more "Edward knows that Sally knows..." clauses there are, Edward and Sally both know that they both know (and so on) that Edward is aware of the car crash.

Bart's sense is that, no matter how many more "Edward knows that Sally knows..." clauses there are, Edward and Sally do not both know that they both know (and so on) that Edward is aware of the car crash.

Hart, however, thinks there is no way to know the answer, since he has no sense at all of whether the sentence could be correct.

5. With whom do you agree more, Art, Bart, or Hart?

- ○ Art
- ○ Bart
- ○ Hart

6. What was the story about?

- ○ An office complaint.
- ○ A car crash.
- ○ A train confrontation.

1_2 - SHARED TRAIN

Edward and Sally are sitting across from each other on a train, when Edward notices a man on the other side of the train silently walk up to another passenger who is wearing a provocative t-shirt, say something to him, then give him the finger.

While this is happening, Sally glances toward Edward and sees that he is watching the event. Edward immediately spots this out of the corner of his eye, but pretends not to notice her.

Put yourself in Edward's shoes. Please rate how certain you are about each of the things that Edward might be aware of. Think carefully about each statement-- it may seem complicated at first, but most people find that when they take their time and think about each sentence in turn, the situation becomes clear. Please consider each one independently of the others.

What is Edward aware of?

1. A train confrontation just happened.

No, he definitely isn't aware of that	Yes, he definitely is aware of that

0 10 20 30 40 50 60 70 80 90 100

2. "Sally knows I'm aware of the train confrontation."

No, he definitely isn't aware of that	Yes, he definitely is aware of that

0 10 20 30 40 50 60 70 80 90 100

3. "Sally knows I'm aware of the train confrontation. And she understands that I realize that she knows it."

No, he definitely isn't aware of that	Yes, he definitely is aware of that

0 10 20 30 40 50 60 70 80 90 100

4. "Sally knows I'm aware of the train confrontation. And she understands that I realize that she knows it, and also that I know that she knows this."

No, he definitely isn't aware of that	Yes, he definitely is aware of that

0 10 20 30 40 50 60 70 80 90 100

Here's the story again:

Edward and Sally are sitting across from each other on a train, when Edward notices a man on the other side of the train silently walk up to another passenger who is wearing a provocative t-shirt, say something to him, then give him the finger.

While this is happening, Sally glances toward Edward and sees that he is watching the event. Edward immediately spots this out of the corner of his eye, but pretends not to notice her.

Now, let's imagine whether Edward is thinking something really complicated:

"Sally knows that I'm aware that she realizes that I understand that she recognizes that I know that she's aware that I realize that she understands that I recognize that she knows that I'm aware that she realizes that I understand that she recognizes that I know that she understands that <u>I'm aware of the train confrontation."</u>

Imagine three people named Art, Bart and Hart. Art, Bart and Hart disagree about their understanding of this sentence. Although they all find it very difficult to think about, Art and Bart think this is irrelevant, since they still know the answer.

Art's sense is that, no matter how many more "Edward knows that Sally knows..." clauses there are, Edward and Sally both know that they both know (and so on) that Edward is aware of the train confrontation.

Bart's sense is that, no matter how many more "Edward knows that Sally knows..." clauses there are, Edward and Sally do not both know that they both know (and so on) that Edward is aware of the train confrontation.

Hart, however, thinks there is no way to know the answer, since he has no sense at all of whether the sentence could be correct.

5. With whom do you agree more, Art, Bart, or Hart?

- ○ **Art**
- ○ **Bart**
- ○ **Hart**

6. What was the story about?

- ○ **An office complaint.**
- ○ **A car crash.**
- ○ **A train confrontation.**

1_3 - PUBLIC OFFICE

Sally barges into her bosses' office, not noticing that her co-worker, Edward, is seated on the couch behind her. She shouts in frustration, "Please tell Edward to get rid of his childish Looney Tunes cellphone ringtone. Yesterday his phone rang in front of an important client.

Right at this point, Edward gets a call and his phone rings loudly, playing the Looney Tunes song.

Put yourself in Edward's shoes. Please rate how certain you are about each of the things that Edward might be aware of. Think carefully about each statement-- it may seem complicated at first, but most people find that when they take their time and think about each sentence in turn, the situation becomes clear. Please consider each one independently of the others.

What is Edward aware of?

1. Sally complained about me.

No, he definitely isn't aware of that — Yes, he definitely is aware of that

0 10 20 30 40 50 60 70 80 90 100

2. "Sally knows I'm aware she complained about me."

No, he definitely isn't aware of that | Yes, he definitely is aware of that

0 10 20 30 40 50 60 70 80 90 100

3. "Sally knows I'm aware she complained about me. And she understands that I realize she knows it."

No, he definitely isn't aware of that | Yes, he definitely is aware of that

0 10 20 30 40 50 60 70 80 90 100

4. "Sally knows I'm aware she complained about me. And she understands that I realize she knows it, and also that I know that she knows this."

No, he definitely isn't aware of that | Yes, he definitely is aware of that

0 10 20 30 40 50 60 70 80 90 100

Here's the story again:

Sally barges into her bosses' office, not noticing that her co-worker, Edward, is seated on the couch behind her. She shouts in frustration, "Please tell Edward to get rid of his childish Looney Tunes cellphone ringtone. Yesterday his phone rang in front of an important client.

Right at this point, Edward gets a call and his phone rings loudly, playing the Looney Tunes song.

Now, let's imagine whether Edward is thinking something really complicated:

"Sally knows that I'm aware that she realizes that I understand that she recognizes that I know that she's aware that I realize that she understands that I recognize that she knows that I'm aware that she realizes that I understand that she recognizes that I know that she understands that I'm aware she complained about me."

Imagine three people named Art, Bart and Hart. Art, Bart and Hart disagree about their understanding of this sentence. Although they all find it very difficult to think about, Art and Bart think this is irrelevant, since they still know the answer.

Art's sense is that, no matter how many more "Edward knows that Sally knows..." clauses there are, Edward and Sally both know that they both know (and so on) that Edward is aware Sally complained about him.

Bart's sense is that, no matter how many more "Edward knows that Sally knows..." clauses there are, Edward and Sally do not both know that they both know (and so on) that Edward is aware Sally complained about him.

Hart, however, thinks there is no way to know the answer, since he has no sense at all of whether the sentence could be correct.

5. With whom do you agree more, Art, Bart, or Hart?

- ○ **Art**
- ○ **Bart**
- ○ **Hart**

6. What was the story about?

- ○ **An office complaint.**
- ○ **A car crash.**
- ○ **A train confrontation.**

2_1 - PRIVATE CRASH

Edward and his neighbor, Sally, share a garden in a medium-sized apartment complex. One day Edward is watering their garden, when a car crash occurs on the street right in front of the garden in which he is working.

Sally’s roommate notices the event from her window, and calls Sally to let her know about the crash and that Edward witnessed it.

Put yourself in Edward's shoes. Please rate how certain you are about each of the things that Edward might be aware of. Think carefully about each statement-- it may seem complicated at first, but most people find that when they take their time and think about each sentence in turn, the

situation becomes clear. Please consider each one independently of the others.

What is Edward aware of?

1. A car crash just happened.

No, he definitely isn't aware of that	Yes, he definitely is aware of that

0 10 20 30 40 50 60 70 80 90 100

2. "Sally knows I'm aware of the car crash."

No, he definitely isn't aware of that	Yes, he definitely is aware of that

0 10 20 30 40 50 60 70 80 90 100

3. "Sally knows I'm aware of the car crash. And she understands that I realize that she knows it."

No, he definitely isn't aware of that | Yes, he definitely is aware of that

0 10 20 30 40 50 60 70 80 90 100

4. "Sally knows I'm aware of the car crash. And she understands that I realize that she knows it, and also that I know that she knows this."

No, he definitely isn't aware of that | Yes, he definitely is aware of that

0 10 20 30 40 50 60 70 80 90 100

Here's the story again:

Edward and his neighbor, Sally, share a garden in a medium-sized apartment complex. One day Edward is alone in the garden watering the plants, when a car crash occurs on the street right in front of the garden in which he is working.

Sally's roommate notices the event from her window, and calls Sally right then to let her know about the crash and that Edward witnessed it.

Now, let's imagine whether Edward is thinking something really complicated:

"Sally knows that I'm aware that she realizes that I understand that she recognizes that I know that she's aware that I realize that she understands that I recognize that she knows that I'm aware that she realizes that I

understand that she recognizes that I know that she understands that I'm aware of the car crash."

Imagine three people named Art, Bart and Hart. Art, Bart and Hart disagree about their understanding of this sentence. Although they all find it very difficult to think about, Art and Bart think this is irrelevant, since they still know the answer.

Art's sense is that, no matter how many more "Edward knows that Sally knows..." clauses there are, Edward and Sally both know that they both know (and so on) that Edward is aware of the car crash.

Bart's sense is that, no matter how many more "Edward knows that Sally knows..." clauses there are, Edward and Sally do not both know that they both know (and so on) that Edward is aware of the car crash.

Hart, however, thinks there is no way to know the answer, since he has no sense at all of whether the sentence could be correct.

5. With whom do you agree more, Art, Bart, or Hart?

- ○ **Art**
- ○ **Bart**
- ○ **Hart**

6. What was the story about?

- ○ **An office complaint.**
- ○ **A car crash.**

○ **A train confrontation.**

2_2 - SHARED OFFICE

Sally barges into her bosses' office and shouts in frustration, "Please tell Edward to get rid of his childish Looney Tunes cellphone ringtone. Yesterday his phone rang in front of an important client".

Just as Sally is uttering this sentence, Edward walks passed the room and overhears her say this. Instead of continuing down the hallway, Edward hides outside the door and hears Sally say, "Oops, I just saw Edward and I think he heard everything".

Put yourself in Edward's shoes. Please rate how certain you are about each of the things that Edward might be aware of. Think carefully about each statement-- it may seem complicated at first, but most people find that when they take their time and think about each sentence in turn, the situation becomes clear. Please consider each one independently of the others.

What is Edward aware of?

1. Sally complained about me.

No, he definitely isn't aware of that | Yes, he definitely is aware of that

0 10 20 30 40 50 60 70 80 90 100

2. "Sally knows I'm aware she complained about me."

No, he definitely isn't aware of that | Yes, he definitely is aware of that

0 10 20 30 40 50 60 70 80 90 100

3. "Sally knows I'm aware she complained about me. And she understands that I realize she knows it."

No, he definitely isn't aware of that | Yes, he definitely is aware of that

0 10 20 30 40 50 60 70 80 90 100

4. "Sally knows I'm aware she complained about me. And she understands that I realize she knows it, and also that I know that she knows this."

No, he definitely isn't aware of that | Yes, he definitely is aware of that

0 10 20 30 40 50 60 70 80 90 100

Here's the story again:

Sally barges into her bosses' office and shouts in frustration, "Please tell Edward to get rid of his childish Looney Tunes cellphone ringtone. Yesterday his phone rang in front of an important client".

Just as Sally is uttering this sentence, Edward walks passed the room and overhears her say this. Instead of continuing down the hallway, Edward hides outside the door and hears Sally say, "Oops, I just saw Edward and I think he heard everything".

Now, let's imagine whether Edward is thinking something really complicated:

"Sally knows that I'm aware that she realizes that I understand that she recognizes that I know that she's aware that I realize that she understands that I recognize that she knows that I'm aware that she realizes that I understand that she recognizes that I know that she understands that <u>I'm aware she complained about me."</u>

Imagine three people named Art, Bart and Hart. Art, Bart and Hart disagree about their understanding of this sentence. Although they all find it very difficult to think about, Art and Bart think this is irrelevant, since they still know the answer.

Art's sense is that, no matter how many more "Edward knows that Sally knows..." clauses there are, Edward and Sally both know that they both know (and so on) that Edward is aware Sally complained about him.

Bart's sense is that, no matter how many more "Edward knows that Sally knows..." clauses there are, Edward and Sally do not both know that they both know (and so on) that Edward is aware Sally complained about him.

Hart, however, thinks there is no way to know the answer, since he has no sense at all of whether the sentence could be correct.

5. With whom do you agree more, Art, Bart, or Hart?

- Art
- Bart
- Hart

6. What was the story about?

- An office complaint.
- A car crash.
- A train confrontation.

2_3 - PUBLIC TRAIN

Edward and Sally are sitting across from each other on a train, when Edward notices a man between them loudly walk up to another passenger

who is wearing a provocative t-shirt, shout something to him, then give him the finger.

Put yourself in Edward's shoes. Please rate how certain you are about each of the things that Edward might be aware of. Think carefully about each statement-- it may seem complicated at first, but most people find that when they take their time and think about each sentence in turn, the situation becomes clear. Please consider each one independently of the others.

What is Edward aware of?

1. A train confrontation just happened.

No, he definitely isn't aware of that | Yes, he definitely is aware of that

0 10 20 30 40 50 60 70 80 90 100

2. "Sally knows I'm aware of the train confrontation."

No, he definitely isn't aware of that | Yes, he definitely is aware of that

0 10 20 30 40 50 60 70 80 90 100

3. "Sally knows I'm aware of the train confrontation. And she understands that I realize that she knows it."

No, he definitely isn't aware of that — Yes, he definitely is aware of that

0 10 20 30 40 50 60 70 80 90 100

4. "Sally knows I'm aware of the train confrontation. And she understands that I realize that she knows it, and also that I know that she knows this."

No, he definitely isn't aware of that — Yes, he definitely is aware of that

0 10 20 30 40 50 60 70 80 90 100

Here's the story again:

Edward and Sally are sitting across from each other on a train, when Edward notices a man between them loudly walk up to another passenger who is wearing a provocative t-shirt, shout something to him, then give him the finger.

Now, let's imagine whether Edward is thinking something really complicated:

"Sally knows that I'm aware that she realizes that I understand that she recognizes that I know that she's aware that I realize that she understands that I recognize that she knows that I'm aware that she realizes that I understand that she recognizes that I know that she understands that I'm aware of the train confrontation."

Imagine three people named Art, Bart and Hart. Art, Bart and Hart disagree about their understanding of this sentence. Although they all find it very difficult to think about, Art and Bart think this is irrelevant, since they still know the answer.

Art's sense is that, no matter how many more "Edward knows that Sally knows..." clauses there are, Edward and Sally both know that they both know (and so on) that Edward is aware of the train confrontation.

Bart's sense is that, no matter how many more "Edward knows that Sally knows..." clauses there are, Edward and Sally do not both know that they both know (and so on) that Edward is aware of the train confrontation.

Hart, however, thinks there is no way to know the answer, since he has no sense at all of whether the sentence could be correct.

5. With whom do you agree more, Art, Bart, or Hart?

- ○ **Art**
- ○ **Bart**
- ○ **Hart**

6. What was the story about?

- An office complaint.
- A car crash.
- A train confrontation.

3_1 - PRIVATE TRAIN

Edward and Sally are sitting across from each other on a train, when Edward notices a man on the other side of the train silently walk up to another passenger who is wearing a provocative t-shirt, say something to him, then give him the finger.

While this is happening, Sally glances toward Edward and sees that he is watching the event. However, Edward does not notice Sally do this.

Put yourself in Edward's shoes. Please rate how certain you are about each of the things that Edward might be aware of. Think carefully about each statement-- it may seem complicated at first, but most people find that when they take their time and think about each sentence in turn, the situation becomes clear. Please consider each one independently of the others.

What is Edward aware of?

1. A train confrontation just happened.

No, he definitely isn't aware of that | Yes, he definitely is aware of that

0 10 20 30 40 50 60 70 80 90 100

2. "Sally knows I'm aware of the train confrontation."

No, he definitely isn't aware of that | Yes, he definitely is aware of that

0 10 20 30 40 50 60 70 80 90 100

3. "Sally knows I'm aware of the train confrontation. And she understands that I realize that she knows it."

No, he definitely isn't aware of that | Yes, he definitely is aware of that

0 10 20 30 40 50 60 70 80 90 100

4. "Sally knows I'm aware of the train confrontation. And she understands that I realize that she knows it, and also that I know that she knows this."

No, he definitely isn't aware of that | Yes, he definitely is aware of that

0 10 20 30 40 50 60 70 80 90 100

Here's the story again:

Edward and Sally are sitting across from each other on a train, when Edward notices a man on the other side of the train silently walk up to another passenger who is wearing a provocative t-shirt, say something to him, then give him the finger.

While this is happening, Sally glances toward Edward and sees that he is watching the event. However, Edward does not notice Sally do this.

Now, let's imagine whether Edward is thinking something really complicated:

"Sally knows that I'm aware that she realizes that I understand that she recognizes that I know that she's aware that I realize that she understands that I recognize that she knows that I'm aware that she realizes that I understand that she recognizes that I know that she understands that I'm aware of the train confrontation."

Imagine three people named Art, Bart and Hart. Art, Bart and Hart disagree about their understanding of this sentence. Although they all find it very difficult to think about, Art and Bart think this is irrelevant, since they still know the answer.

Art's sense is that, no matter how many more "Edward knows that Sally knows..." clauses there are, Edward and Sally both know that they both know (and so on) that Edward is aware of the train confrontation.

Bart's sense is that, no matter how many more "Edward knows that Sally knows..." clauses there are, Edward and Sally do not both know that they both know (and so on) that Edward is aware of the train confrontation.

Hart, however, thinks there is no way to know the answer, since he has no sense at all of whether the sentence could be correct.

5. With whom do you agree more, Art, Bart, or Hart?

- ○ **Art**
- ○ **Bart**
- ○ **Hart**

6. What was the story about?

- ○ **An office complaint.**
- ○ **A car crash.**
- ○ **A train confrontation.**

3_2 - SHARED CRASH

Edward and his neighbor, Sally, share a garden in a medium-sized apartment complex. One day Edward is watering their garden, when a car

crash occurs on the street right in front of the garden in which he is working.

Sally's roommate notices the event from her window, and calls Sally to let her know about the crash and that Edward witnessed it. She then goes outside to talk to Edward about the crash, and tells him all about her phone call with Sally.

Put yourself in Edward's shoes. Please rate how certain you are about each of the things that Edward might be aware of. Think carefully about each statement-- it may seem complicated at first, but most people find that when they take their time and think about each sentence in turn, the situation becomes clear. Please consider each one independently of the others.

What is Edward aware of?

1. A car crash just happened.

No, he definitely isn't aware of that						Yes, he definitely is aware of that				
0	10	20	30	40	50	60	70	80	90	100

2. "Sally knows I'm aware of the car crash."

No, he definitely isn't aware of that | Yes, he definitely is aware of that

0 10 20 30 40 50 60 70 80 90 100

3. "Sally knows I'm aware of the car crash. And she understands that I realize that she knows it."

No, he definitely isn't aware of that | Yes, he definitely is aware of that

0 10 20 30 40 50 60 70 80 90 100

4. "Sally knows I'm aware of the car crash. And she understands that I realize that she knows it, and also that I know that she knows this."

No, he definitely isn't aware of that | Yes, he definitely is aware of that

0 10 20 30 40 50 60 70 80 90 100

Here's the story again:

Edward and his neighbor, Sally, share a garden in a medium-sized apartment complex. One day Edward is watering their garden, when a car crash occurs on the street right in front of the garden in which he is working.

Sally's roommate notices the event from her window, and calls Sally to let her know about the crash and that Edward witnessed it. She then goes outside to talk to Edward about the crash, and tells him all about her phone call with Sally.

Now, let's imagine whether Edward is thinking something really complicated:

"Sally knows that I'm aware that she realizes that I understand that she recognizes that I know that she's aware that I realize that she understands that I recognize that she knows that I'm aware that she realizes that I understand that she recognizes that I know that she understands that I'm aware of the car crash."

Imagine three people named Art, Bart and Hart. Art, Bart and Hart disagree about their understanding of this sentence. Although they all find it very difficult to think about, Art and Bart think this is irrelevant, since they still know the answer.

Art's sense is that, no matter how many more "Edward knows that Sally knows..." clauses there are, Edward and Sally both know that they both know (and so on) that Edward is aware of the car crash.

Bart's sense is that, no matter how many more "Edward knows that Sally knows..." clauses there are, Edward and Sally do not both know that they both know (and so on) that Edward is aware of the car crash.

Hart, however, thinks there is no way to know the answer, since he has no sense at all of whether the sentence could be correct.

5. With whom do you agree more, Art, Bart, or Hart?

- Art
- Bart
- Hart

6. What was the story about?

- An office complaint.
- A car crash.
- A train confrontation.

3_3 - PUBLIC OFFICE

Sally barges into her bosses' office, not noticing that her co-worker, Edward, is seated on the couch behind her. She shouts in frustration, "Please tell Edward to get rid of his childish Looney Tunes cellphone ringtone. Yesterday his phone rang in front of an important client.

Right at this point, Edward gets a call and his phone rings loudly, playing the Looney Tunes song.

Put yourself in Edward's shoes. Please rate how certain you are about each of the things that Edward might be aware of. Think carefully about each statement-- it may seem complicated at first, but most people find that when they take their time and think about each sentence in turn, the situation becomes clear. Please consider each one independently of the others.

What is Edward aware of?

1. Sally complained about me.

No, he definitely isn't aware of that — Yes, he definitely is aware of that

0 10 20 30 40 50 60 70 80 90 100

2. "Sally knows I'm aware she complained about me."

No, he definitely isn't aware of that — Yes, he definitely is aware of that

0 10 20 30 40 50 60 70 80 90 100

3. "Sally knows I'm aware she complained about me. And she understands that I realize she knows it."

No, he definitely isn't aware of that — Yes, he definitely is aware of that

0 10 20 30 40 50 60 70 80 90 100

4. "Sally knows I'm aware she complained about me. And she understands that I realize she knows it, and also that I know that she knows this."

No, he definitely isn't aware of that	Yes, he definitely is aware of that

0 10 20 30 40 50 60 70 80 90 100

Here's the story again:

Sally barges into her bosses' office, not noticing that her co-worker, Edward, is seated on the couch behind her. She shouts in frustration, "Please tell Edward to get rid of his childish Looney Tunes cellphone ringtone. Yesterday his phone rang in front of an important client.

Right at this point, Edward gets a call and his phone rings loudly, playing the Looney Tunes song.

Now, let's imagine whether Edward is thinking something really complicated:

"Sally knows that I'm aware that she realizes that I understand that she recognizes that I know that she's aware that I realize that she understands that I recognize that she knows that I'm aware that she realizes that I understand that she recognizes that I know that she understands that I'm aware she complained about me."

Imagine three people named Art, Bart and Hart. Art, Bart and Hart disagree about their understanding of this sentence. Although they all find it very difficult to think about, Art and Bart think this is irrelevant, since they still know the answer.

Art's sense is that, no matter how many more "Edward knows that Sally knows..." clauses there are, Edward and Sally both know that they both know (and so on) that Edward is aware Sally complained about him.

Bart's sense is that, no matter how many more "Edward knows that Sally knows..." clauses there are, Edward and Sally do not both know that they both know (and so on) that Edward is aware Sally complained about him.

Hart, however, thinks there is no way to know the answer, since he has no sense at all of whether the sentence could be correct.

5. With whom do you agree more, Art, Bart, or Hart?

- ○ **Art**
- ○ **Bart**
- ○ **Hart**

6. What was the story about?

- ○ **An office complaint.**
- ○ **A car crash.**
- ○ **A train confrontation.**

4_1 - PRIVATE TRAIN

Edward and Sally are sitting across from each other on a train, when Edward notices a man on the other side of the train silently walk up to another passenger who is wearing a provocative t-shirt, say something to him, then give him the finger.

While this is happening, Sally glances toward Edward and sees that he is watching the event. However, Edward does not notice Sally do this.

Put yourself in Edward's shoes. Please rate how certain you are about each of the things that Edward might be aware of. Think carefully about each statement-- it may seem complicated at first, but most people find that when they take their time and think about each sentence in turn, the situation becomes clear. Please consider each one independently of the others.

What is Edward aware of?

1. A train confrontation just happened.

No, he definitely isn't aware of that	Yes, he definitely is aware of that

0 10 20 30 40 50 60 70 80 90 100

2. "Sally knows I'm aware of the train confrontation."

No, he definitely isn't aware of that | Yes, he definitely is aware of that

0 10 20 30 40 50 60 70 80 90 100

3. "Sally knows I'm aware of the train confrontation. And she understands that I realize that she knows it."

No, he definitely isn't aware of that | Yes, he definitely is aware of that

0 10 20 30 40 50 60 70 80 90 100

4. "Sally knows I'm aware of the train confrontation. And she understands that I realize that she knows it, and also that I know that she knows this."

No, he definitely isn't aware of that | Yes, he definitely is aware of that

0 10 20 30 40 50 60 70 80 90 100

Here's the story again:

Edward and Sally are sitting across from each other on a train, when Edward notices a man on the other side of the train silently walk up to

another passenger who is wearing a provocative t-shirt, say something to him, then give him the finger.

While this is happening, Sally glances toward Edward and sees that he is watching the event. However, Edward does not notice Sally do this.

Now, let's imagine whether Edward is thinking something really complicated:

"Sally knows that I'm aware that she realizes that I understand that she recognizes that I know that she's aware that I realize that she understands that I recognize that she knows that I'm aware that she realizes that I understand that she recognizes that I know that she understands that I'm aware of the train confrontation."

Imagine three people named Art, Bart and Hart. Art, Bart and Hart disagree about their understanding of this sentence. Although they all find it very difficult to think about, Art and Bart think this is irrelevant, since they still know the answer.

Art's sense is that, no matter how many more "Edward knows that Sally knows..." clauses there are, Edward and Sally both know that they both know (and so on) that Edward is aware of the train confrontation.

Bart's sense is that, no matter how many more "Edward knows that Sally knows..." clauses there are, Edward and Sally do not both know that they both know (and so on) that Edward is aware of the train confrontation.

Hart, however, thinks there is no way to know the answer, since he has no sense at all of whether the sentence could be correct.

5. With whom do you agree more, Art, Bart, or Hart?

- ○ **Art**
- ○ **Bart**
- ○ **Hart**

6. What was the story about?

- ○ **An office complaint.**
- ○ **A car crash.**
- ○ **A train confrontation.**

4_2 - SHARED OFFICE

Sally barges into her bosses' office and shouts in frustration, "Please tell Edward to get rid of his childish Looney Tunes cellphone ringtone. Yesterday his phone rang in front of an important client".

Just as Sally is uttering this sentence, Edward walks passed the room and overhears her say this. Instead of continuing down the hallway, Edward hides outside the door and hears Sally say, "Oops, I just saw Edward and I think he heard everything".

Put yourself in Edward's shoes. Please rate how certain you are about each of the things that Edward might be aware of. Think carefully about each statement-- it may seem complicated at first, but most people find that when they take their time and think about each sentence in turn, the

situation becomes clear. Please consider each one independently of the others.

What is Edward aware of?

1. Sally complained about me.

No, he definitely isn't aware of that						Yes, he definitely is aware of that				
0	10	20	30	40	50	60	70	80	90	100

2. "Sally knows I'm aware she complained about me."

No, he definitely isn't aware of that						Yes, he definitely is aware of that				
0	10	20	30	40	50	60	70	80	90	100

3. "Sally knows I'm aware she complained about me. And she understands that I realize she knows it."

No, he definitely isn't aware of that | Yes, he definitely is aware of that

0 10 20 30 40 50 60 70 80 90 100

4. "Sally knows I'm aware she complained about me. And she understands that I realize she knows it, and also that I know that she knows this."

No, he definitely isn't aware of that | Yes, he definitely is aware of that

0 10 20 30 40 50 60 70 80 90 100

Here's the story again:

Sally barges into her bosses' office and shouts in frustration, "Please tell Edward to get rid of his childish Looney Tunes cellphone ringtone. Yesterday his phone rang in front of an important client".

Just as Sally is uttering this sentence, Edward walks passed the room and overhears her say this. Instead of continuing down the hallway, Edward hides outside the door and hears Sally say, "Oops, I just saw Edward and I think he heard everything".

Now, let's imagine whether Edward is thinking something really complicated:

"Sally knows that I'm aware that she realizes that I understand that she recognizes that I know that she's aware that I realize that she understands that I recognize that she knows that I'm aware that she realizes that I understand that she recognizes that I know that she understands that I'm aware she complained about me."

Imagine three people named Art, Bart and Hart. Art, Bart and Hart disagree about their understanding of this sentence. Although they all find it very difficult to think about, Art and Bart think this is irrelevant, since they still know the answer.

Art's sense is that, no matter how many more "Edward knows that Sally knows..." clauses there are, Edward and Sally both know that they both know (and so on) that Edward is aware Sally complained about him.

Bart's sense is that, no matter how many more "Edward knows that Sally knows..." clauses there are, Edward and Sally do not both know that they both know (and so on) that Edward is aware Sally complained about him.

Hart, however, thinks there is no way to know the answer, since he has no sense at all of whether the sentence could be correct.

5. With whom do you agree more, Art, Bart, or Hart?

- ○ **Art**
- ○ **Bart**
- ○ **Hart**

6. What was the story about?

- An office complaint.
- A car crash.
- A train confrontation.

4_3 - PUBLIC CRASH

Edward and his neighbor, Sally, share a garden in a medium-sized apartment complex. One day they are talking and watering their garden, when a car crash occurs on the street right in front of the garden in which they are working.

Put yourself in Edward's shoes. Please rate how certain you are about each of the things that Edward might be aware of. Think carefully about each statement-- it may seem complicated at first, but most people find that when they take their time and think about each sentence in turn, the situation becomes clear. Please consider each one independently of the others.

What is Edward aware of?

1. A car crash just happened.

No, he definitely isn't aware of that | Yes, he definitely is aware of that

0 10 20 30 40 50 60 70 80 90 100

2. "Sally knows I'm aware of the car crash."

No, he definitely isn't aware of that | Yes, he definitely is aware of that

0 10 20 30 40 50 60 70 80 90 100

3. "Sally knows I'm aware of the car crash. And she understands that I realize that she knows it."

No, he definitely isn't aware of that | Yes, he definitely is aware of that

0 10 20 30 40 50 60 70 80 90 100

4. "Sally knows I'm aware of the car crash. And she understands that I realize that she knows it, and also that I know that she knows this."

No, he definitely isn't aware of that	Yes, he definitely is aware of that

0 10 20 30 40 50 60 70 80 90 100

Here's the story again:

Edward and his neighbor, Sally, share a garden in a medium-sized apartment complex. One day they are talking and watering their garden, when a car crash occurs on the street right in front of the garden in which they are working.

Now, let's imagine whether Edward is thinking something really complicated:

"Sally knows that I'm aware that she realizes that I understand that she recognizes that I know that she's aware that I realize that she understands that I recognize that she knows that I'm aware that she realizes that I understand that she recognizes that I know that she understands that I'm aware of the car crash."

Imagine three people named Art, Bart and Hart. Art, Bart and Hart disagree about their understanding of this sentence. Although they all find it very difficult to think about, Art and Bart think this is irrelevant, since they still know the answer.

Art's sense is that, no matter how many more "Edward knows that Sally knows..." clauses there are, Edward and Sally both know that they both know (and so on) that Edward is aware of the car crash.

Bart's sense is that, no matter how many more "Edward knows that Sally knows..." clauses there are, Edward and Sally do not both know that they both know (and so on) that Edward is aware of the car crash.

Hart, however, thinks there is no way to know the answer, since he has no sense at all of whether the sentence could be correct.

5. With whom do you agree more, Art, Bart, or Hart?

- ○ **Art**
- ○ **Bart**
- ○ **Hart**

6. What was the story about?

- ○ **An office complaint.**
- ○ **A car crash.**
- ○ **A train confrontation.**

5_1 - PRIVATE OFFICE

Sally barges into her bosses' office and shouts in frustration, "Please tell Edward to get rid of his childish Looney Tunes cellphone ringtone. Yesterday his phone rang in front of an important client".

Just as Sally is uttering this sentence, Edward walks passed the room and overhears her say this. However, Sally does not see Edward pass by.

Put yourself in Edward's shoes. Please rate how certain you are about each of the things that Edward might be aware of. Think carefully about each statement-- it may seem complicated at first, but most people find that when they take their time and think about each sentence in turn, the situation becomes clear. Please consider each one independently of the others.

What is Edward aware of?

1. Sally complained about me.

No, he definitely isn't aware of that | Yes, he definitely is aware of that

0 10 20 30 40 50 60 70 80 90 100

2. "Sally knows I'm aware she complained about me."

No, he definitely isn't aware of that | Yes, he definitely is aware of that

0 10 20 30 40 50 60 70 80 90 100

3. "Sally knows I'm aware she complained about me. And she understands that I realize that she knows it."

No, he definitely isn't aware of that | Yes, he definitely is aware of that

0 10 20 30 40 50 60 70 80 90 100

4. "Sally knows I'm aware she complained about me. And she understands that I realize that she knows it, and also that I know that she knows this."

No, he definitely isn't aware of that | Yes, he definitely is aware of that

0 10 20 30 40 50 60 70 80 90 100

Here's the story again:

Sally barges into her bosses' office and shouts in frustration, "Please tell Edward to get rid of his childish Looney Tunes cellphone ringtone. Yesterday his phone rang in front of an important client".

Just as Sally is uttering this sentence, Edward walks passed the room and overhears her say this. However, Sally does not see Edward pass by.

Now, let's imagine whether Edward is thinking something really complicated:

"Sally knows that I'm aware that she realizes that I understand that she recognizes that I know that she's aware that I realize that she understands that I recognize that she knows that I'm aware that she realizes that I

understand that she recognizes that I know that she understands that I'm aware she complained about me."

Imagine three people named Art, Bart and Hart. Art, Bart and Hart disagree about their understanding of this sentence. Although they all find it very difficult to think about, Art and Bart think this is irrelevant, since they still know the answer.

Art's sense is that, no matter how many more "Edward knows that Sally knows..." clauses there are, Edward and Sally both know that they both know (and so on) that Edward is aware Sally complained about him.

Bart's sense is that, no matter how many more "Edward knows that Sally knows..." clauses there are, Edward and Sally do not both know that they both know (and so on) that Edward is aware Sally complained about him.

Hart, however, thinks there is no way to know the answer, since he has no sense at all of whether the sentence could be correct.

5. With whom do you agree more, Art, Bart, or Hart?

- ○ **Art**
- ○ **Bart**
- ○ **Hart**

6. What was the story about?

- ○ **An office complaint.**

- **A car crash.**
- **A train confrontation.**

5_2 - SHARED CRASH

Edward and his neighbor, Sally, share a garden in a medium-sized apartment complex. One day Edward is watering their garden, when a car crash occurs on the street right in front of the garden in which he is working.

Sally's roommate notices the event from her window, and calls Sally to let her know about the crash and that Edward witnessed it. She then goes outside to talk to Edward about the crash, and tells him all about her phone call with Sally.

Put yourself in Edward's shoes. Please rate how certain you are about each of the things that Edward might be aware of. Think carefully about each statement-- it may seem complicated at first, but most people find that when they take their time and think about each sentence in turn, the situation becomes clear. Please consider each one independently of the others.

What is Edward aware of?

1. A car crash just happened.

No, he definitely isn't aware of that | Yes, he definitely is aware of that

0 10 20 30 40 50 60 70 80 90 100

2. "Sally knows I'm aware of the car crash."

No, he definitely isn't aware of that | Yes, he definitely is aware of that

0 10 20 30 40 50 60 70 80 90 100

3. "Sally knows I'm aware of the car crash. And she understands that I realize that she knows it."

No, he definitely isn't aware of that | Yes, he definitely is aware of that

0 10 20 30 40 50 60 70 80 90 100

4. "Sally knows I'm aware of the car crash. And she understands that I realize that she knows it, and also that I know that she knows this."

No, he definitely isn't aware of that						Yes, he definitely is aware of that				
0	10	20	30	40	50	60	70	80	90	100

Here's the story again:

Edward and his neighbor, Sally, share a garden in a medium-sized apartment complex. One day Edward is watering their garden, when a car crash occurs on the street right in front of the garden in which he is working.

Sally's roommate notices the event from her window, and calls Sally to let her know about the crash and that Edward witnessed it. She then goes outside to talk to Edward about the crash, and tells him all about her phone call with Sally.

Now, let's imagine whether Edward is thinking something really complicated:

"Sally knows that I'm aware that she realizes that I understand that she recognizes that I know that she's aware that I realize that she understands that I recognize that she knows that I'm aware that she realizes that I understand that she recognizes that I know that she understands that I'm aware of the car crash."

Imagine three people named Art, Bart and Hart. Art, Bart and Hart disagree about their understanding of this sentence. Although they

all find it very difficult to think about, Art and Bart think this is irrelevant, since they still know the answer.

Art's sense is that, no matter how many more "Edward knows that Sally knows..." clauses there are, Edward and Sally both know that they both know (and so on) that Edward is aware of the car crash.

Bart's sense is that, no matter how many more "Edward knows that Sally knows..." clauses there are, Edward and Sally do not both know that they both know (and so on) that Edward is aware of the car crash.

Hart, however, thinks there is no way to know the answer, since he has no sense at all of whether the sentence could be correct.

5. With whom do you agree more, Art, Bart, or Hart?

- ○ Art
- ○ Bart
- ○ Hart

6. What was the story about?

- ○ An office complaint.
- ○ A car crash.
- ○ A train confrontation.

5_3 - PUBLIC TRAIN

Edward and Sally are sitting across from each other on a train, when Edward notices a man between them loudly walk up to another passenger who is wearing a provocative t-shirt, shout something to him, then give him the finger.

Put yourself in Edward's shoes. Please rate how certain you are about each of the things that Edward might be aware of. Think carefully about each statement-- it may seem complicated at first, but most people find that when they take their time and think about each sentence in turn, the situation becomes clear. Please consider each one independently of the others.

What is Edward aware of?

1. A train confrontation just happened.

No, he definitely isn't aware of that | Yes, he definitely is aware of that

0 10 20 30 40 50 60 70 80 90 100

2. "Sally knows I'm aware of the train confrontation."

No, he definitely isn't aware of that | Yes, he definitely is aware of that

0 10 20 30 40 50 60 70 80 90 100

3. "Sally knows I'm aware of the train confrontation. And she understands that I realize that she knows it."

No, he definitely isn't aware of that | Yes, he definitely is aware of that

0 10 20 30 40 50 60 70 80 90 100

4. "Sally knows I'm aware of the train confrontation. And she understands that I realize that she knows it, and also that I know that she knows this."

No, he definitely isn't aware of that | Yes, he definitely is aware of that

0 10 20 30 40 50 60 70 80 90 100

Here's the story again:

Edward and Sally are sitting across from each other on a train, when Edward notices a man between them loudly walk up to another passenger

who is wearing a provocative t-shirt, shout something to him, then give him the finger.

Now, let's imagine whether Edward is thinking something really complicated:

"Sally knows that I'm aware that she realizes that I understand that she recognizes that I know that she's aware that I realize that she understands that I recognize that she knows that I'm aware that she realizes that I understand that she recognizes that I know that she understands that <u>I'm aware of the train confrontation."</u>

Imagine three people named Art, Bart and Hart. Art, Bart and Hart disagree about their understanding of this sentence. Although they all find it very difficult to think about, Art and Bart think this is irrelevant, since they still know the answer.

Art's sense is that, no matter how many more "Edward knows that Sally knows..." clauses there are, Edward and Sally both know that they both know (and so on) that Edward is aware of the train confrontation.

Bart's sense is that, no matter how many more "Edward knows that Sally knows..." clauses there are, Edward and Sally <u>do not</u> both know that they both know (and so on) that Edward is aware of the train confrontation.

Hart, however, thinks there is no way to know the answer, since he has no sense at all of whether the sentence could be correct.

5. With whom do you agree more, Art, Bart, or Hart?

- Art
- Bart
- Hart

6. What was the story about?

- An office complaint.
- A car crash.
- A train confrontation.

6_1 - PRIVATE OFFICE

Sally barges into her bosses' office and shouts in frustration, "Please tell Edward to get rid of his childish Looney Tunes cellphone ringtone. Yesterday his phone rang in front of an important client".

Just as Sally is uttering this sentence, Edward walks passed the room and overhears her say this. However, Sally does not see Edward pass by.

Put yourself in Edward's shoes. Please rate how certain you are about each of the things that Edward might be aware of. Think carefully about each statement-- it may seem complicated at first, but most people find that when they take their time and think about each sentence in turn, the situation becomes clear. Please consider each one independently of the others.

What is Edward aware of?

1. Sally complained about me.

No, he definitely isn't aware of that | Yes, he definitely is aware of that

0 10 20 30 40 50 60 70 80 90 100

2. "Sally knows I'm aware she complained about me."

No, he definitely isn't aware of that | Yes, he definitely is aware of that

0 10 20 30 40 50 60 70 80 90 100

3. "Sally knows I'm aware she complained about me. And she understands that I realize that she knows it."

No, he definitely isn't aware of that | Yes, he definitely is aware of that

0 10 20 30 40 50 60 70 80 90 100

4. "Sally knows I'm aware she complained about me. And she understands that I realize that she knows it, and also that I know

that she knows this."

No, he definitely isn't aware of that						Yes, he definitely is aware of that				
0	10	20	30	40	50	60	70	80	90	100

Here's the story again:

Sally barges into her bosses' office and shouts in frustration, "Please tell Edward to get rid of his childish Looney Tunes cellphone ringtone. Yesterday his phone rang in front of an important client".

Just as Sally is uttering this sentence, Edward walks passed the room and overhears her say this. However, Sally does not see Edward pass by.

Now, let's imagine whether Edward is thinking something really complicated:

"Sally knows that I'm aware that she realizes that I understand that she recognizes that I know that she's aware that I realize that she understands that I recognize that she knows that I'm aware that she realizes that I understand that she recognizes that I know that she understands that I'm aware she complained about me."

Imagine three people named Art, Bart and Hart. Art, Bart and Hart disagree about their understanding of this sentence. Although they all find it very difficult to think about, Art and Bart think this is irrelevant, since they still know the answer.

Art's sense is that, no matter how many more "Edward knows that Sally knows..." clauses there are, Edward and Sally both know that they both know (and so on) that Edward is aware Sally complained about him.

Bart's sense is that, no matter how many more "Edward knows that Sally knows..." clauses there are, Edward and Sally do not both know that they both know (and so on) that Edward is aware Sally complained about him.

Hart, however, thinks there is no way to know the answer, since he has no sense at all of whether the sentence could be correct.

5. With whom do you agree more, Art, Bart, or Hart?

- Art
- Bart
- Hart

6. What was the story about?

- An office complaint.
- A car crash.
- A train confrontation.

6_2 - SHARED TRAIN

Edward and Sally are sitting across from each other on a train, when Edward notices a man on the other side of the train silently walk up to

another passenger who is wearing a provocative t-shirt, say something to him, then give him the finger.

While this is happening, Sally glances toward Edward and sees that he is watching the event. Edward immediately spots this out of the corner of his eye, but pretends not to notice her.

Put yourself in Edward's shoes. Please rate how certain you are about each of the things that Edward might be aware of. Think carefully about each statement-- it may seem complicated at first, but most people find that when they take their time and think about each sentence in turn, the situation becomes clear. Please consider each one independently of the others.

What is Edward aware of?

1. A train confrontation just happened.

No, he definitely isn't aware of that — Yes, he definitely is aware of that

0 10 20 30 40 50 60 70 80 90 100

2. "Sally knows I'm aware of the train confrontation."

No, he definitely isn't aware of that						Yes, he definitely is aware of that				
0	10	20	30	40	50	60	70	80	90	100

3. "Sally knows I'm aware of the train confrontation. And she understands that I realize that she knows it."

No, he definitely isn't aware of that						Yes, he definitely is aware of that				
0	10	20	30	40	50	60	70	80	90	100

4. "Sally knows I'm aware of the train confrontation. And she understands that I realize that she knows it, and also that I know that she knows this."

No, he definitely isn't aware of that						Yes, he definitely is aware of that				
0	10	20	30	40	50	60	70	80	90	100

Here's the story again:

Edward and Sally are sitting across from each other on a train, when Edward notices a man on the other side of the train silently walk up to

another passenger who is wearing a provocative t-shirt, say something to him, then give him the finger.

While this is happening, Sally glances toward Edward and sees that he is watching the event. Edward immediately spots this out of the corner of his eye, but pretends not to notice her.

Now, let's imagine whether Edward is thinking something really complicated:

"Sally knows that I'm aware that she realizes that I understand that she recognizes that I know that she's aware that I realize that she understands that I recognize that she knows that I'm aware that she realizes that I understand that she recognizes that I know that she understands that I'm aware of the train confrontation."

Imagine three people named Art, Bart and Hart. Art, Bart and Hart disagree about their understanding of this sentence. Although they all find it very difficult to think about, Art and Bart think this is irrelevant, since they still know the answer.

Art's sense is that, no matter how many more "Edward knows that Sally knows..." clauses there are, Edward and Sally both know that they both know (and so on) that Edward is aware of the train confrontation.

Bart's sense is that, no matter how many more "Edward knows that Sally knows..." clauses there are, Edward and Sally do not both know that they both know (and so on) that Edward is aware of the train confrontation.

Hart, however, thinks there is no way to know the answer, since he has no sense at all of whether the sentence could be correct.

5. With whom do you agree more, Art, Bart, or Hart?

- ○ **Art**
- ○ **Bart**
- ○ **Hart**

6. What was the story about?

- ○ **An office complaint.**
- ○ **A car crash.**
- ○ **A train confrontation.**

6_3 - PUBLIC CRASH

Edward and his neighbor, Sally, share a garden in a medium-sized apartment complex. One day they are talking and watering their garden, when a car crash occurs on the street right in front of the garden in which they are working.

Put yourself in Edward's shoes. Please rate how certain you are about each of the things that Edward might be aware of. Think carefully about each statement-- it may seem complicated at first, but most people find that when they take their time and think about each sentence in turn, the situation becomes clear. Please consider each one independently of the others.

What is Edward aware of?

1. A car crash just happened.

No, he definitely isn't aware of that	Yes, he definitely is aware of that

0 10 20 30 40 50 60 70 80 90 100

2. "Sally knows I'm aware of the car crash."

No, he definitely isn't aware of that	Yes, he definitely is aware of that

0 10 20 30 40 50 60 70 80 90 100

3. "Sally knows I'm aware of the car crash. And she understands that I realize that she knows it."

No, he definitely isn't aware of that	Yes, he definitely is aware of that

0 10 20 30 40 50 60 70 80 90 100

4. "Sally knows I'm aware of the car crash. And she understands that I realize that she knows it, and also that I know that she knows this."

No, he definitely isn't aware of that	Yes, he definitely is aware of that

0 10 20 30 40 50 60 70 80 90 100

Here's the story again:

Edward and his neighbor, Sally, share a garden in a medium-sized apartment complex. One day they are talking and watering their garden, when a car crash occurs on the street right in front of the garden in which they are working.

Now, let's imagine whether Edward is thinking something really complicated:

"Sally knows that I'm aware that she realizes that I understand that she recognizes that I know that she's aware that I realize that she understands that I recognize that she knows that I'm aware that she realizes that I understand that she recognizes that I know that she understands that I'm aware of the car crash."

Imagine three people named Art, Bart and Hart. Art, Bart and Hart disagree about their understanding of this sentence. Although they all find it very difficult to think about, Art and Bart think this is irrelevant, since they still know the answer.

Art's sense is that, no matter how many more "Edward knows that Sally knows..." clauses there are, Edward and Sally both know that they both know (and so on) that Edward is aware of the car crash.

Bart's sense is that, no matter how many more "Edward knows that Sally knows..." clauses there are, Edward and Sally do not both know that they both know (and so on) that Edward is aware of the car crash.

Hart, however, thinks there is no way to know the answer, since he has no sense at all of whether the sentence could be correct.

5. With whom do you agree more, Art, Bart, or Hart?

- Art
- Bart
- Hart

6. What was the story about?

- An office complaint.
- A car crash.
- A train confrontation.

COMPREHENSION & DEMOGRAPHICS

What is your gender?

- Male

- Female
- Prefer not to disclose
- Other

What is your ethnicity?

- White
- Black
- Asian
- Mixed
- Other

What is your age (in years)?

Please indicate the highest level of education completed.

- Grammar School
- High School or Equivalent
- Vocational/Technical School (2 year)
- Some College
- College Graduate (4 year)
- Masters Degree (MS)

- Doctoral Degree (PhD)
- Professional Degree (MD, JD, etc.)
- Other

Any comments on the survey?

DEBRIEFING

Debriefing: Judgments and Visual Processing

1. What was this study about?
The aim of the current study is to explore how what we see influences the commonsense judgments we make. We are interested in how your brain gets from pixels impinging on your retina to something as complex as a moral judgment.

2. How was the study conducted?
In today's study you were asked to view some videos or images and then answer some questionnaires that contained questions about thoughts, feelings, and personality attributes. Your physiological responses may have also been recorded while you viewed stimuli.

3. What was the hypothesis?
We expect to find that specific features that we manipulate in our displays, e.g., the amount of contact between two objects, or the delay between when one object touches another and when the second moves, will systematically influence people's judgments.

4. Did we tell you everything?
Yes. There was no deception involved in this study.

5. Why is this study important?
What is the relationship between the things we see and the judgments we make? People who study vision tend to think of it as determining why we see what we do, but they don't often think of it as influencing the kinds of everyday judgments we make. Yet some more recent work (for a review, see Scholl & Tremoulet, 2000; Gao, McCarthy, & Scholl, 2010) has begun to suggest that even simple dynamic displays consisting only of simple shapes can automatically give rise to much richer notions, such as whether something is alive, responsible, or has a particular goal in mind. Thus, it may be that even simple visual information is intimately involved in our everyday intuitions about things and the eventual judgments we make. For example, it may be that even simple shapes moving around can even give rise to the sense of something being right or wrong, which then determines the kinds of moral judgments we make.

The purpose of this study is to investigate the relationship between visual processing and everyday intuitions and judgments, e.g., between vision and morality, or between vision and the self.

6. References:
Gao, T., McCarthy, G., & Scholl, B. J. (2010). The wolfpack effect: Perception of animacy irresistibly influences interactive behavior. Psychological Science, 21(12), 1845-1853.

Scholl, B. J., & Tremoulet, P. (2000). Perceptual causality and animacy. Trends in Cognitive Sciences, 4(8), 299-309.

7. How to contact the researcher: If you have questions or concerns about your participation or payment, or want to request a summary of research findings, please contact the researcher: Julian De Freitas, PhD

candidate, 700 William James Hall, 33 Kirkland St, Cambridge, MA 02138; defreitas@g.harvard.edu. For any problems related to this study, you may also contact the faculty member who is supervising it: George A. Alvarez, alvarez@wjh.harvard.edu.

8. Whom to contact about your right as a participant in this research.

For questions, concerns, suggestions, or complaints that have not been or cannot be addressed by the researcher, or to report research-related harm, please contact the Committee on the Use of Human Subjects in Research at Harvard University, 44-R Brattle Street, Suite 200, Cambridge, MA 02138. Email: cuhs@harvard.edu

Powered by Qualtrics

For study 2, the Qualtrics PDF export cannot display the embedded video stimuli. The videos presented to participants are therefore provided below for completeness:

Private Information condition Video: https://youtu.be/v3c9_kQz6SY
Reciprocal Information condition Video: https://youtu.be/MCFgM_v2RJg
Doubly Reciprocal Information Condition Video: https://youtu.be/XVjK8AmZk1E
Public Information Condition Video: https://youtu.be/VXq-bAD6CiA

Questions

CONSENT

Participation is voluntary

It is your choice whether or not to participate in this research. If you choose to participate, you may change your mind and leave the study at any time. Refusal to participate or stopping your participation will involve no penalty or loss of benefits to which you are otherwise entitled.

What is the purpose of this research?

The purpose of this research is to examine human visual performance and judgments. All data from this experiment are gathered for scientific purposes and will contribute to our eventual understanding of brain and visual function. These data may be published in scientific journals so that other researchers may have access to these data.

How long will I take part in this research?

Your participation will take approximately 7 minutes to complete.

What can I expect if I take part in this research?

As a participant, you will be asked to look at images presented on a video display and give responses with key presses or movements of a mouse pointing device. Your response may involve responding as quickly as you can, memorizing what you saw, making a judgment, or completing a questionnaire. You will also be asked to complete a demographics form.

What are the risks and possible discomforts? If you choose to participate, the effects should be comparable to those you would experience from viewing a computer monitor for 7 minutes and using a

mouse or keyboard, e.g., eye fatigue. You are free to take breaks throughout the session. Some of the images and stories are mildly emotional, and some of the written stories are disgust-inducing.

Will I be compensated for participating in this research?

You will be compensated $1 for this study. You will still receive payment if you withdraw early.

If I take part in this research, how will my privacy be protected? What happens to the information you collect?

Your participation in this experiment will remain confidential, and your identity will not be stored with your data.

If I have any questions, concerns or complaints about this research study, who can I talk to?

The researcher for this study is Julian De Freitas who can be reached at #161 Morgan Hall, 15 Harvard Way, Boston MA, 02163; jdefreitas@hbs.edu. If you have questions, concerns, or complaints, If you would like to talk to the research team, If you think the research has harmed you, or If you wish to withdraw from the study.

This research has been reviewed by the Committee on the Use of Human Subjects in Research at Harvard University. They can be reached at 617-496-2847, 1350 Massachusetts Avenue, Suite 935, Cambridge, MA 02138, or cuhs@harvard.edu for any of the following: If your questions, concerns, or complaints are not being answered by the research team, If you cannot reach the research team, If you want to talk to someone besides the research team, or If you have questions about your rights as a research participant.

Do you consent?

- ◯ Yes
- ◯ No

Prolific ID

What is your Prolific ID?

[ ]

ATTENTION CHECKS

John is taller than Paul. Who is shorter?

- ○ John
- ○ Paul
- ○ Neither John nor Paul
- ○ Both John and Paul

What color is grass?

The fresh, uncut grass, not leaves or hay. Please make sure to select purple, so that we know you're paying attention.

- ○ Green
- ○ Purple

ATTENTION CHECKS 2

You failed one of the previous attention checks. Please pay closer attention!
Here are some more attention checks:

This is an attention check. Please set X at 15, and make it so Y is larger than X, and Z is larger than Y. Please also make sure that Y is evenly divisible by 10.

0 10 20 30 40 50 60 70 80 90 100

X

Y

Z

How many <u>fatal</u> heart attacks have you had?

- 0
- 1
- 10

- 100
- 1000

INTRO

Welcome! You will watch **Four** short videos and answer questions about each one. Please watch them **very carefully**.

Private condition_A

In this video, you will see a university library, in which food and drink are prohibited. A student at the end of the table has broken the rule by bringing in a cup of coffee, and he has spilled it on the table. There are two security guards between the bookshelves, Edward and Sally (Please note that there is no audio in this video, and please assume that Ed and Sally never talk to each other about the incident after it happens.):

Private Information condition Video: https://youtu.be/v3c9_kQz6SY

0:00 / 0:10

What is Edward aware of?

(Please feel free to consult the video again at the bottom of this page)

1. "Someone spilled a cup of coffee in an area where food and drink are prohibited."

No, he definitely would not think that. | Yes, he could very well think that.

0 10 20 30 40 50 60 70 80 90 100

2. "Sally knows I'm aware of the coffee incident."

No, he definitely would not think that. | Yes, he could very well think that.

0 10 20 30 40 50 60 70 80 90 100

3. "Sally knows I'm aware of the coffee incident. And she understands that I realize that she knows that I'm aware of the coffee incident."

No, he definitely would not think that. | Yes, he could very well think that.

0 10 20 30 40 50 60 70 80 90 100

4. "Sally knows I'm aware of the incident. And she understands that I realize that she knows that I'm aware of the coffee incident, and also that I know that she knows this."

No, he definitely would not think that. | Yes, he could very well think that.

0 10 20 30 40 50 60 70 80 90 100

Private Information condition Video: https://youtu.be/v3c9_kQz6SY

0:00 / 0:10

Now, let's imagine whether Edward is thinking something really complicated: **"Sally knows that I'm aware that she realizes that I understand that she recognizes that I know that she's aware that I realize that she understands that I recognize that she knows that I'm aware that she realizes that I understand that she recognizes that I know that she understands that <u>I'm aware of the coffee incident.</u>"**

Imagine three people named **Art, Bart** and **Hart**. Art, Bart and Hart disagree about their understanding of this sentence.

Although they all find it very difficult to think about, Art and Bart think this is irrelevant, since they still know the answer.

- Art's sense is that, no matter how many more "Edward knows that Sally knows..." clauses there are, Edward and Sally both know that they both know (and so on) that Edward is aware of the coffee incident.
- Bart's sense is that, no matter how many more "Edward knows that Sally knows..." clauses there are, Edward and Sally do not both know that they both know (and so on) that Edward is aware of the coffee incident.
- Hart, however, thinks there is no way to know the answer, since he has no sense at all of whether the sentence could be correct.

5. With whom do you agree more, **Art, Bart,** or **Hart**?

- ○ Art
- ○ Bart
- ○ Hart

transition_1to2

Now you will watch the **second** video. It is different from the **first** one. Please watch it carefully before answering questions.

Secondary condition_B

In this video, you will see a university library, in which food and drink are prohibited. A student at the end of the table has broken the rule by bringing in a cup of coffee, and he has spilled it on the table. There are two security guards between the bookshelves, Edward and Sally (Please note that there is no audio in this video, and please assume that Ed and Sally never talk to each other about the incident after it happens.):

Reciprocal Information condition Video: https://youtu.be/MCFgM_v2RJg

0:00 / 0:27

What is Edward aware of?

(Please feel free to consult the video again at the bottom of this page)

1. “Someone spilled a cup of coffee in an area where food and drink are prohibited.”

No, he definitely would not think that. | Yes, he could very well think that.

0 10 20 30 40 50 60 70 80 90 100

2. "Sally knows I'm aware of the coffee incident."

No, he definitely would not think that. | Yes, he could very well think that.

0 10 20 30 40 50 60 70 80 90 100

3. "Sally knows I'm aware of the coffee incident. And she understands that I realize that she knows that I'm aware of the coffee incident."

No, he definitely would not think that. | Yes, he could very well think that.

0 10 20 30 40 50 60 70 80 90 100

4. "Sally knows I'm aware of the incident. And she understands that I realize that she knows that I'm aware of the coffee incident, and also that I know that she knows this."

No, he definitely would not think that. | Yes, he could very well think that.

0 10 20 30 40 50 60 70 80 90 100

Reciprocal Information condition Video: https://youtu.be/MCFgM_v2RJg

0:00 / 0:27

Now, let's imagine whether Edward is thinking something really complicated: **"Sally knows that I'm aware that she realizes that I understand that she recognizes that I know that she's aware that I realize that she understands that I recognize that she knows that I'm aware that she realizes that I understand that she recognizes that I know that she understands that <u>I'm aware of the coffee incident</u>."**

Imagine three people named **Art, Bart** and **Hart**. Art, Bart and Hart disagree about their understanding of this sentence. Although they all find it very difficult to think about, Art and

Bart think this is irrelevant, since they still know the answer.

- Art's sense is that, no matter how many more "Edward knows that Sally knows..." clauses there are, Edward and Sally both know that they both know (and so on) that Edward is aware of the coffee incident.
- Bart's sense is that, no matter how many more "Edward knows that Sally knows..." clauses there are, Edward and Sally do not both know that they both know (and so on) that Edward is aware of the coffee incident.
- Hart, however, thinks there is no way to know the answer, since he has no sense at all of whether the sentence could be correct.

5. With whom do you agree more, **Art, Bart,** or **Hart**?

- Art
- Bart
- Hart

transition_2to3

Now you will watch the **third** video. It is different from the **second** one. Please watch it carefully before answering questions.

# Shared condition_C

In this video, you will see a university library, in which food and drink are prohibited. A student at the end of the table has broken the rule by bringing in a cup of coffee, and he has spilled it on the table. There are two security guards between the bookshelves, Edward and Sally (Please note that there is no audio in this video, and please assume that Ed and Sally never talk to each other about the incident after it happens.):

Doubly Reciprocal Information Condition Video: https://youtu.be/XVjK8AmZk1E

0:00 / 1:34

What is Edward aware of?

(Please feel free to consult the video again at the bottom of this page)

1. “Someone spilled a cup of coffee in an area where food and drink are prohibited.”

No, he definitely would not think that. Yes, he could very well think that.

0 10 20 30 40 50 60 70 80 90 100

2. "Sally knows I'm aware of the coffee incident."

No, he definitely would not think that. Yes, he could very well think that.

0 10 20 30 40 50 60 70 80 90 100

3. "Sally knows I'm aware of the coffee incident. And she understands that I realize that she knows that I'm aware of the coffee incident."

No, he definitely would not think that. Yes, he could very well think that.

0 10 20 30 40 50 60 70 80 90 100

4. "Sally knows I'm aware of the incident. And she understands that I realize that she knows that I'm aware of the coffee incident, and also that I know that she knows this."

No, he definitely would not think that. Yes, he could very well think that.

0 10 20 30 40 50 60 70 80 90 100

Doubly Reciprocal Information Condition Video: https://youtu.be/XVjK8AmZk1E

0:00 / 1:34

Now, let's imagine whether Edward is thinking something really complicated: **"Sally knows that I'm aware that she realizes that I understand that she recognizes that I know that she's aware that I realize that she understands that I recognize that she knows that I'm aware that she realizes that I understand that she recognizes that I know that she understands that I'm aware of the coffee incident."**

Imagine three people named **Art, Bart** and **Hart**. Art, Bart and Hart disagree about their understanding of this sentence. Although they all find it very difficult to think about, Art and Bart think this is irrelevant, since they still know the answer.

- Art's sense is that, no matter how many more "Edward knows that Sally knows..." clauses there are, Edward and Sally both know that they both know (and so on) that Edward is aware of the coffee incident.
- Bart's sense is that, no matter how many more "Edward knows that Sally knows..." clauses there are, Edward and Sally do not both know that they both know (and so on) that Edward is aware of the coffee incident.
- Hart, however, thinks there is no way to know the answer, since he has no sense at all of whether the sentence could be correct.

5. With whom do you agree more, **Art, Bart,** or **Hart**?

- ○ Art
- ○ Bart
- ○ Hart

transition_3to4

Now you will watch the **last video**. It is different from the **third** one. Please watch it carefully before answering questions.

Public condition_D

In this video, you will see a university library, in which food and drink are prohibited. A student at the end of the table has broken the rule by bringing in a cup of coffee, and he has spilled it on the table. There are two security guards between the bookshelves, Edward and Sally (Please note that there is no audio in this video, and please assume that Ed and Sally never talk to each other about the incident after it happens.):

Public Information Condition Video: https://youtu.be/VXq-bAD6CiA

0:00 / 0:14

What is Edward aware of?

(Please feel free to consult the video again at the bottom of this page)

1. "Someone spilled a cup of coffee in an area where food and drink are prohibited."

No, he definitely would not think that. — Yes, he could very well think that.

0 10 20 30 40 50 60 70 80 90 100

2. "Sally knows I'm aware of the coffee incident."

No, he definitely would not think that. | Yes, he could very well think that.

0 10 20 30 40 50 60 70 80 90 100

3. "Sally knows I'm aware of the coffee incident. And she understands that I realize that she knows that I'm aware of the coffee incident."

No, he definitely would not think that. | Yes, he could very well think that.

0 10 20 30 40 50 60 70 80 90 100

4. "Sally knows I'm aware of the incident. And she understands that I realize that she knows that I'm aware of the coffee incident, and also that I know that she knows this."

No, he definitely would not think that. | Yes, he could very well think that.

0 10 20 30 40 50 60 70 80 90 100

Public Information Condition Video: https://youtu.be/VXq-bAD6CiA

0:00 / 0:14

Now, let's imagine whether Edward is thinking something really complicated: **"Sally knows that I'm aware that she realizes that I understand that she recognizes that I know that she's aware that I realize that she understands that I recognize that she knows that I'm aware that she realizes that I understand that she recognizes that I know that she understands that I'm aware of the coffee incident."**

Imagine three people named **Art, Bart** and **Hart.** Art, Bart and Hart disagree about their understanding of this sentence. Although they all find it very difficult to think about, Art and

Bart think this is irrelevant, since they still know the answer.

- Art's sense is that, no matter how many more "Edward knows that Sally knows..." clauses there are, Edward and Sally both know that they both know (and so on) that Edward is aware of the coffee incident.
- Bart's sense is that, no matter how many more "Edward knows that Sally knows..." clauses there are, Edward and Sally do not both know that they both know (and so on) that Edward is aware of the coffee incident.
- Hart, however, thinks there is no way to know the answer, since he has no sense at all of whether the sentence could be correct.

5. With whom do you agree more, **Art, Bart,** or **Hart?**

- ○ Art
- ○ Bart
- ○ Hart

check

What were the videos about?

- ○ A train confrontation.
- ○ A coffee incident.

COMPREHENSION & DEMOGRAPHICS

What is your gender?

- Male
- Female
- Prefer not to disclose
- Other

What is your ethnicity?

- White
- Black
- Asian
- Mixed
- Other

What is your age (in years)?

Please indicate the highest level of education completed.

- Grammar School
- High School or Equivalent
- Vocational/Technical School (2 year)
- Some College
- College Graduate (4 year)
- Masters Degree (MS)
- Doctoral Degree (PhD)
- Professional Degree (MD, JD, etc.)
- Other

Any comments on the survey?

DEBRIEFING

Debriefing: Common knowledge and public event

1. What was this study about?

The aim of the current study is to explore how what we see influences the commonsense judgments we make. We are interested in how your brain generate recursive knowledge about other people in different events.

2. How was the study conducted?

In today's study you were asked to view some videos and then answer some questionnaires that contained questions about recursive knowledge and how easy the mentalizing process was. You were presented with one of the four conditions regarding the publicity of an event among the witnesses. We intend to test how people represent common knowledge. One possibility is that people represent it as a public event. It is a follow up study in a larger project: study 1 was carried our in language scenarios, whereas study 2, the current study, provides a generalization and external validity test by using custom video scenarios with minimal language description.

3. What was the hypothesis?

We expect to find that people will make an inferential leap to common knowledge when the event is public—and only when it is public. Even though people cannot actually represent infinite nestings, they grasp when they effectively have infinite beliefs.

4. Did we tell you everything?

Yes. There was no deception involved in this study.

5. Why is this study important?

Understanding how people represent common knowledge is crucial for advancing our comprehension of human social cognition and communication. Common knowledge underpins many social interactions, cooperative behaviors, and collective decision-making processes.

6. How to contact the researcher: If you have questions or concerns about your participation or payment, or want to request a summary of research findings, please contact the researcher: [Dr. Julian De Freitas, 33 Kirkland St, Cambridge, MA 02138; defreitas@g.harvard.edu.]

7. Whom to contact about your right as a participant in this research.

For questions, concerns, suggestions, or complaints that have not been or cannot be addressed by the researcher, or to report research-related harm, please contact the Committee on the Use of Human Subjects in Research at Harvard University, 44-R Brattle

Street, Suite 200, Cambridge, MA 02138. Email: cuhs@harvard.edu

Prolific code: CDZ3EJOX

Powered by Qualtrics
